# Fear learning in unmedicated patients with anxiety disorders: a comparison of delay conditioning, fear reversal, and trace conditioning

**DOI:** 10.1038/s41398-026-03996-6

**Published:** 2026-04-02

**Authors:** Enric Vilajosana, Simone Battaglia, Pamela Chavarría-Elizondo, Ignacio Martínez‑Zalacaín, Asier Juaneda-Seguí, Cristina Saiz-Masvidal, Víctor De la Peña-Arteaga, Alexander J. Shackman, Joaquim Radua, Carles Soriano-Mas, Miquel A. Fullana

**Affiliations:** 1 Institut d’Investigacions Biomèdiques August Pi i Sunyer, Barcelona, Spain; 2 Department of Medicine, Institute of Neurosciences, University of Barcelona, Barcelona, Spain; 3 University of Vic – Central University of Catalonia, C. Sagrada Família, 7, 08500 Vic Barcelona, Spain; 4 Department of Theoretical and Applied Sciences, eCampus University, Novedrate (Como), Italy; 5 Psychiatry and Mental Health Group, Neuroscience Program, Institut d’Investigació Biomèdica de Bellvitge (IDIBELL), L’Hospitalet de Llobregat, Barcelona, Spain; 6 Department of Clinical Sciencies, Universitat de Barcelona, Barcelona, Spain; 7 Network Center for Biomedical Research on Mental Health (CIBERSAM), Instituto de Salud Carlos III (ISCIII), Madrid, Spain; 8 Radiology Department, Hospital Universitari de Bellvitge, L’Hospitalet de Llobregat, Carrer de Feixa Llarga SN, 08907 Barcelona, Spain; 9 Sant Pau Mental Health Research Group, Institut de Recerca Sant Pau (IR SANT PAU), Barcelona, Spain; 10 Department of Psychology, University of Maryland, College Park, MD 20742 US; 11 Neuroscience and Cognitive Science Program, University of Maryland, College Park, MD 20742 US; 12 Maryland Neuroimaging Center, University of Maryland, College Park, MD 20742 US; 13 Department of Social Psychology and Quantitative Psychology, Institute of Neurosciences, Universitat de Barcelona (UB), and Institut d’Investigació Biomèdica de Bellvitge (IDIBELL) and CIBERSAM, Barcelona, Spain; 14 Adult Psychiatry and Psychology Department, Institute of Neurosciences, Hospital Clinic, Barcelona, Spain

**Keywords:** Psychiatric disorders, Human behaviour, Learning and memory

## Abstract

Anxiety disorders are common and impairing, yet their underlying mechanisms remain incompletely understood. Fear learning provides a critical translational framework for investigating pathological anxiety, bridging laboratory models and clinical phenomena. Prior studies have been limited by important methodological issues, including the inclusion of non-anxiety diagnoses, high comorbidity, and medication use. Here we examined three forms of fear learning— delay conditioning, fear reversal, and trace conditioning—in unmedicated adults with minimally comorbid primary anxiety disorders (Generalized Anxiety Disorder and Social Anxiety Disorder; n = 34) and demographically matched controls (n = 102). Individuals with anxiety disorders showed greater psychophysiological arousal (skin conductance responses) and reduced brain activation (assessed using functional magnetic resonance imaging) in the left dorsolateral prefrontal cortex to the learned safety cue (CS − ) during the early phase of delay conditioning. Differences between individuals with anxiety disorders and controls were not evident for the learned threat versus learned safety (CS+ versus CS − ) contrasts during delay conditioning, fear-reversal, or trace conditioning in psychophysiological arousal, brain activation, or subjective ratings. Taken together, these observations underscore the selectivity of Pavlovian learning deficits among unmedicated individuals with anxiety disorders and highlight differences in learning or using safety-related information to adaptively regulate fear.

## Introduction

Anxiety disorders, including generalized anxiety disorder (GAD), social anxiety disorder (SAD) and panic disorder (PD), affect about 12% of adults globally each year, with evidence suggesting that prevalence may be on the rise. [1, 2] These disorders can severely disrupt daily functioning, including occupational and social functioning, relationships, and overall quality of life. [3, 4] Given their impact, understanding the underlying mechanisms of anxiety disorders is essential for developing more effective or tolerable treatments. [2–4]

Pavlovian fear (or threat) learning paradigms have become a crucial translational tool in anxiety disorder research, bridging the gap between laboratory research and clinical practice. [5] These paradigms can be leveraged to study a variety of processes, including the acquisition (hereafter referred to as conditioning), and reversal of learned fears. In fear conditioning, a formerly neutral stimulus elicits fear (conditioned stimulus, CS + ) after being associated with an innately aversive stimulus (unconditioned stimulus, US). Two key forms of Pavlovian conditioning have been characterized. In delay conditioning, the presentation of the CS+ and US overlap in time, with the US typically co-terminating with the cue (i.e., delayed). In trace conditioning, the CS+ and US are separated by a brief interval, requiring the learner to hold a ‘trace’ of the CS in memory. In human research, responses to the CS+ are typically compared to a second cue which, because it is unpaired, is indicative of safety and remains comparatively neutral (CS-). In fear reversal, the contingencies are reversed, requiring individuals to inhibit their learned responses to previously learned threat and safety signals. [6] Across these diverse paradigms, fear responses are typically assessed using a mixture of subjective ratings; psychophysiological responses, such as the skin conductance response (SCR); and neuroimaging measures, such as functional magnetic resonance imaging (fMRI). [7, 8] Delay conditioning is associated with higher arousal and more negative valence ratings, increased SCRs, and greater activation in regions of the salience / central autonomic–interoceptive network (e.g., anterior insula, dorsal anterior cingulate cortex, thalamus, and sensory cortices) when comparing CS+ to CS − . [7, 9] Trace conditioning shows a similar pattern in subjective ratings and SCRs, but could be additionally characterized by increased hippocampal activation. [10] During fear reversal, subjective ratings and SCRs “flip” to follow the new CS + , and neural activity shifts accordingly: salience-network regions track the new CS + , while regions such as the ventromedial prefrontal cortex and orbitofrontal cortex contribute to flexible re-learning. [6, 11]

Both fear conditioning and fear reversal processes may play a crucial role in anxiety disorders. Increased susceptibility to conditioning (e.g., heightened fear responses to neutral stimuli) may explain persistent fear associations in patients with anxiety disorders. Conversely, impaired fear reversal may reflect difficulty adapting to changing cues, such as failing to respond to new threats or overreacting to now-safe stimuli. [12] Flexible updating of threat associations is also key to effective treatment of anxiety disorders. [13]

Studies assessing fear conditioning and fear reversal in individuals with anxiety disorders have yielded inconsistent findings. A comprehensive recent meta-analysis of delay*-*conditioning paradigms found no consistent differences in threat (CS + ) reactivity among individuals with mixed anxiety and trauma diagnoses, as indexed by psychophysiological responses (SCR). [14] Nevertheless, patients did show heightened responses to safety cues (CS-) across multiple measures, including fear-potentiated startle, US expectancy, and affective ratings, suggesting aberrant safety learning rather than heightened fear conditioning. [14] While an important advance, these observations are limited by the inclusion of medicated individuals. Current anti-anxiety medications can have significant effects on fear learning-including safety learning- processes. [15] Moreover, this meta-analysis -and previous similar work [16] - combined individuals with a wide variety of disorders that are not currently classified as anxiety disorders (e.g., obsessive–compulsive disorder [OCD] or post-traumatic stress disorder [PTSD]). [17] The meta-analysis also did not account for comorbidity, which is a critical inferential limitation given that approximately 60% of individuals with an anxiety disorder also meet criteria for a depressive or other anxiety disorder. [2]

Fear reversal in anxiety disorders remains understudied. In one of the few published studies, Savage and colleagues reported no significant differences in ratings, psychophysiological arousal, or brain activation measures during reversal between unmedicated young patients (aged 15–25) with SAD and healthy controls. [18] In a predominantly medicated GAD sample, Roberts et al. found that those patients had a significantly higher overall SCR and a reduced differential SCR (CS + > CS-) compared to healthy controls during the early, but not the late, phase of fear reversal. [15]

The study by Roberts et al. underscored the importance of temporal dynamics in human fear learning. For example, previous research suggests that learning during fear acquisition is typically stronger in early trials than in later ones. [19] In neuroimaging studies, early trials of fear acquisition are thought to more effectively capture the activation of specific brain regions. [9] Moreover, theoretical and computational models suggest that the largest prediction error—and therefore the greatest amount of learning—occurs when the CS–US contingency is first introduced, i.e., during the early trials. [20] Finally, besides the study by Roberts et al, several previous fear learning studies have found patient-control differences only in early or late learning phases. [21, 22]

Trace fear conditioning has received even less empirical attention, and no prior research has specifically investigated trace conditioning in individuals with anxiety disorders. This is unfortunate because trace paradigms may better reflect real-life situations where cues and aversive outcomes are temporally separated [23] and are considered “weak” situations compared to the “strong” delay paradigms. [24, 25] Weakening the situation, by reducing the certainty, proximity, or intensity of the US, may enhance sensitivity to group differences. [24]

To address these fundamental questions, the present study investigated delay conditioning, fear reversal, and trace conditioning in an unmedicated sample of 34 adults with DSM-5 [17] anxiety diagnoses (primarily GAD or SAD), with minimal or no comorbidity, and 102 age and gender-matched controls. Consistent with recent recommendations, [7] we acquired a comprehensive set of fear measures, including subjective ratings, SCR, and fMRI. Based on previous research, [14, 15] we anticipated that individuals with anxiety disorders would show 1) heightened responses to the CS− during delay conditioning, indicating impaired safety learning; 2) reduced differential conditioning during fear reversal, reflecting difficulties in updating threat and safety associations; and heightened fear conditioning or deficient safety learning during delay conditioning. Based on previous research (see above), we generally expected group differences to be more evident during the earlier portion of each learning phase (e.g., early delay acquisition).

## Methods

### Participants

Participants were recruited as part of a larger study focused on identifying predictors of pathological anxiety. Here, we investigated potential differences in Pavlovian fear conditioning in unmedicated individuals with anxiety disorders (n = 34) and healthy controls (n = 102), selected from a larger sample (n = 135). The two groups were matched on gender distribution and age (Table 1). Diagnostic eligibility was determined by an experienced clinician using the MINI International Neuropsychiatric Interview. [26] For descriptive purposes, participants completed self-reported measures of anxiety, depressive symptoms, and dispositional negative affect (see “**Recruitment procedures**” and “**Self-report measures**” in Sup. Mat.). All participants provided informed written consent. The study was approved by the ethics committee at Hospital de Bellvitge in Barcelona (protocol # PR144/16).

**Table 1:** Demographic and clinical characteristics of participants.

Variable	Healthy Controls (n = *102*) *Mean (SD)*	Patients with Anxiety Disorders (n = *34*) *Mean (SD)*	*Significance*
**Age**	25.6 (4.82)	25.6 (3.8)	*n.s*.
**Females (n, %)**	57 (55.9%)	19 (55.9%)	*n.s*.
**Self-report questionnaires**			
STAI-T (0–60)	18.68 (9.66)	29.15 (11.8)	*p* < 0.001
IUS (27–135)	50.74 (15.56)	72.68 (25.76)	*p* < 0.001
LSAS (0–144)	22.23 (12.39)	32.47 (17.54)	*p* < 0.05
GAD-Screening Scale (0–12)	2.3 (2.13)	6.53 (3.14)	*p* < 0.001
PSWQ-11 (11–55)	25.72 (9.4)	36.41 (10.08)	*p* < 0.001
DASS-S (0–21)	3.41 (2.98)	7.09 (4.23)	*p* < 0.001
DASS-A (0–21)	1.38 (1.88)	3.47 (3.29)	*p* < 0.05
DASS-D (0-21)	1.77 (2.03)	4.88 (4.76)	*p* < 0.001
**Shock aversiveness** ^a^ (1–10)	9.32 (0.84)	9.35 (0.72)	*n.s*.
**Diagnoses**		**Number of participants (%)**	
GAD		24 (70.6)	
GAD plus another anxiety disorder		4 (11.8)^b^	
SAD		5 (14.7)	
SAD plus agoraphobia		1 (2.9)	

*STAI-T* state-trait anxiety inventory, trait version, *IUS* intolerance of uncertainty scale, *LSAS* liebowitz social anxiety scale, *GAD-Screening* generalized anxiety disorder – screening scale, *PSWQ-11* penn state worry questionnaire, *DASS-21-S* depression, anxiety and stress scales – stress subscale, *DASS-21-A* depression, anxiety and stress scales – anxiety subscale, *DASS-21-D* depression, anxiety and stress scale – depression subscale, *GAD* generalized anxiety disorder, *SAD* social anxiety disorder, *n.s.* non-significant.

^a^Average shock aversiveness for the two tasks (see Supplementary Methods).

^b^GAD+Panic Disorder (n = 1); GAD + SAD (n = 3)

### Fear learning assessment

Participants completed two fear-learning tasks in the scanner while subjective ratings, SCR, and fMRI were assessed. The first task assessed delay fear conditioning and fear reversal, whereas the second task assessed trace fear conditioning. The order of the tasks was counterbalanced across participants. In both tasks, the unconditioned stimulus (US) was an individually calibrated electric shock, designed to be “unpleasant but not painful”. In the delay/reversal task, the conditioned stimuli (CSs) were blue and yellow spheres presented against a black background, whereas in the trace task, the CSs were waves, dots, or triangles. Both tasks used the same procedures for subjective ratings, SCR, and fMRI data collection, and participants received identical instructions (see “**Fear learning assessment**” in the Sup. Mat.).

### Delay fear conditioning and fear reversal task

**Fig. 1:**
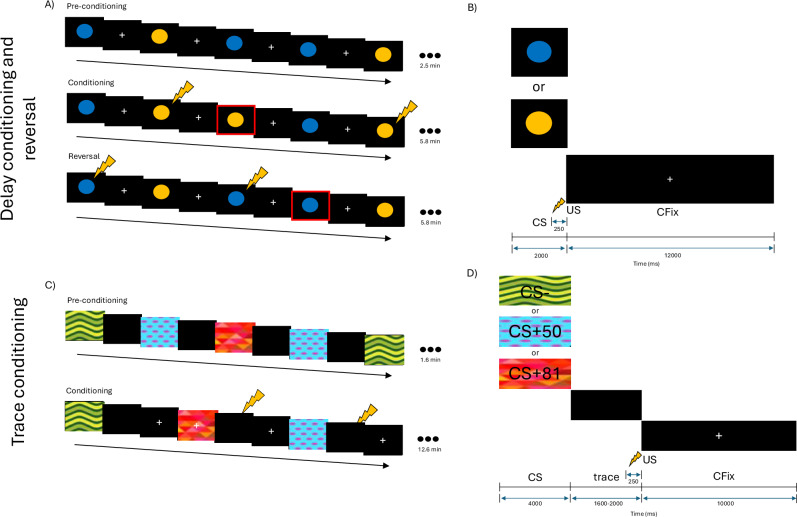
Fear conditioning fMRI paradigms. Delay fear conditioning and fear reversal task. During pre-conditioning, the US was omitted. During conditioning, the US (*lightning bolt*) was paired with one of the spheres on 33.3% trials (CS + ), but not the other (CS-). During fear reversal, the CS-shock contingency was reversed to create newCS+ and newCS-. Red boxes indicate unpaired CS+ trials (**A**). Detailed timeline of events within delay conditioning/reversal trials (**B**). Trace fear conditioning task. During pre-conditioning, the US was omitted. During conditioning, the US was paired with two of the CS (CS + 50: 50%; CS + 81: 81%), but not the third (CS-) (**C**). Detailed timeline of events within trace conditioning trials (**D**). CFix Cross-fixation, CS conditioned stimulus, ms milliseconds, US unconditioned stimulus.

We leveraged a previously validated delay fear acquisition/reversal task that encompassed three phases: pre-conditioning, fear conditioning, and fear reversal [27] (Fig. 1A, B). During pre-conditioning, the to-be-conditioned CS+ and CS- (2000 ms) were each presented five times. The US (250 ms) was never presented. During conditioning, the CS+ and US co-terminated on one-third of trials, enabling us to examine skin-conductance and fMRI responses unconfounded by US presentation. The CS- was never paired with the US. During fear reversal, the CS-shock contingency was reversed (newCS + : *p* = 33.3%; newCS-: *p* = 0.0%). Across the conditioning and reversal phases, there were a total of 15 CS + /newCS+ trials (5 reinforced) and 10 CS-/newCS- trials (pseudorandomized). During the conditioning phase, the second CS+ trial was reinforced. During the reversal phase, the first presentation of the new CS+ was reinforced. CS stimuli were counterbalanced across participants. Across all phases, the inter-trial interval (ITI) between CS trials was 12 s, during which a white fixation cross (CFix) was presented.

### Trace fear conditioning task

The trace conditioning task encompassed two phases: pre-conditioning (baseline), and trace fear conditioning (Fig. 1C, D). During preconditioning, two to-be-conditioned CS+ and one CS- were each presented twice. The US (250 ms) was never presented. During conditioning, the US was presented at the end of the “trace” (blank screen), 1600-2000 ms following CS+ offset. One of the CS+ was reinforced on 50% of trials (CS + 50) and the other was reinforced on 81% of trials (CS + 81). Procedures for the CS- were similar, but it was never paired with the US. During the conditioning phase, each of the three CS was presented 16 times (order pseudorandomized; 11.6-12.0-s ITI). CS stimuli were counterbalanced across participants.

### Measures of conditioned fear

#### Subjective ratings

Immediately after each learning phase (pre-conditioning, conditioning, and reversal for the delay/reversal task, and pre-conditioning and conditioning for the trace conditioning task), participants rated each CS on two five-point Likert scales of valence and arousal related to anxiety (Self-Assessment Manikins [28]), with higher scores indicating greater valence and increased arousal (see “**Measures of conditioned fear**” in the Sup. Mat.).

### Skin conductance responses

SCR data were acquired in the scanner during the two tasks, and the response to each CS (CS + , CS-, for the delay task; newCS+ and newCS- for the reversal task; and CS + 81, CS + 50, and CS-, for the trace task) calculated. The acquisition and (pre)processing of SCR data followed standard procedures. [29] (see **“Measures of conditioned fear”** in the Sup. Mat.**)**.

### Brain activation

Neuroimaging data were acquired using a Phillips Ingenia 3 T scanner (32-channel head-coil). For details on imaging acquisition and (pre)processing, see **“Measures of conditioned fear** - **Brain activation**” in the Sup. Mat.

The two fear learning tasks were programmed in E-Prime 2.0 and displayed on an MRI-compatible back-projection screen. Both tasks were similar in duration (~16 min) and separated by a 15 min break.

*First-level fMRI modeling*. Each participant’s preprocessed time series was entered into a first-level general linear model (GLM) analysis. The onsets of each CS event type were modeled separately for each task by convolving them with a canonical hemodynamic response function. Six motion parameters were included as nuisance covariates. For the delay task, contrast images were computed for CS + > CS− (excluding reinforced trials to avoid contamination from the US) and CS − > CFix. Fixation-cross ITIs contributed the implicit baseline. For the reversal task, contrast images were estimated for newCS + > newCS − , also excluding reinforced trials. For the trace conditioning task, contrasts were computed for CS + ₅₀ > CS − , CS + ₈₁ > CS − , and CS + ₅₀ > CS + ₈₁ and all CS+ trials were included as increasing the ITI and ISI (inter-stimuli interval) minimized the risk of US-related confounds. ISIs ranged from 5.35–5.75 s.

### Statistical analyses

Two-sample Student’s *t*-tests and a chi-square test were used to confirm that patients and controls were adequately matched on demographic characteristics and differed in self-report measures of anxiety. Repeated-measures tests were used to confirm the absence of significant differences between the to-be-conditioned CSs during the preconditioning phase of the delay (Student’s t-tests) and trace (ANOVA) conditioning tasks (see **“Preconditioning Analyses”** in Sup. Mat).

Subjective ratings and SCR data were analyzed using a series of mixed-model ANOVAs with CS as a within-subject factor and group as a between-subject factor. For the acquisition phase of the delay task, there were 2 levels of CS (CS + , CS-). For the reversal phase, there were 2 levels of the CS (newCS + , newCS-). For the trace conditioning task, there were 3 levels of the CS (CS + 50, CS + 81, CS-). For the delay task, the 5 reinforced CS + /newCS+ trials were censored from SCR analyses to avoid US confounding. For the trace task, where a longer CS-US interval prevented US confounding, all trials were included. Post hoc comparisons were conducted using the Bonferroni test (α = 0.05), and the Greenhouse-Geisser correction was applied when necessary. Effect sizes are reported using partial eta squared (η^2^_p_).

Neuroimaging analyses closely paralleled the approach used for SCR. Between-group differences in neuroimaging contrasts (CS + > CS- and CS->CFix for delay conditioning; newCs + >newCS- for fear reversal; CS + 81 > CS-, CS + 50 > CS-, and CS + 81 > CS + 50 for trace conditioning) were assessed using two-sample *t*-tests. Whole-brain statistical significance was determined using a cluster-level family-wise error (FWE) correction at *p* < 0.05, with clusters formed of contiguous voxels with *p* < 0.001.

Consistent with other recent work, [7, 19, 21, 22, 30] we generally expected group differences to be more evident during the early portion of each learning phase. Therefore, we computed a second set of ‘disaggregated’ SCR and fMRI analyses that incorporated early-versus-late phase as a within-subject factor. For the delay and reversal tasks, early and late phases were defined as the first and last five unreinforced CS + /newCS+ and CS-/newCS- trials, respectively. For trace conditioning, they were defined as the first and last eight trials of CS + 81, CS + 50, and CS-. Note that disaggregated analyses for subjective ratings were not possible because these ratings were collected at the end of the phase.

We repeated all main analyses of subjective ratings, SCR, and fMRI data, including age and gender as covariates. Although task order (first delay/reversal or first trace) was counterbalanced, to assess potential order effects, we also repeated the main analyses with task order included as a factor.

## Results

Our patient sample (n = 34) included 28 individuals with a primary diagnosis of GAD and 6 individuals with a primary diagnosis of SAD. There were no significant differences in age or biological sex distribution between patients and controls. Patients exhibited significantly higher anxiety, depressive symptoms, and dispositional negative affect. Groups did not differ in the perceived aversiveness of the shock US (Table 1).

During preconditioning of the delay conditioning/reversal task, no significant differences were observed within each group in responses to the to-be CS+ and to-be CS− across any conditioned fear measures, including subjective ratings, SCR, or brain activation (see Supplementary Fig. 1 and Supplementary Table 2). Similarly, during preconditioning of the trace conditioning task, no significant differences were found in arousal and valence ratings for either group or SCR for the patient group. However, in the control group, SCR responses were greater for the to-be CS + 81 compared to both to-be CS + 50 and to-be CS− (see Supplementary Fig. 2 and Supplementary Table 3). Additionally, both groups exhibited increased activation in the visual cortex in the CS + 81 > CS− contrast.

### Delay fear conditioning

**Fig. 2:**
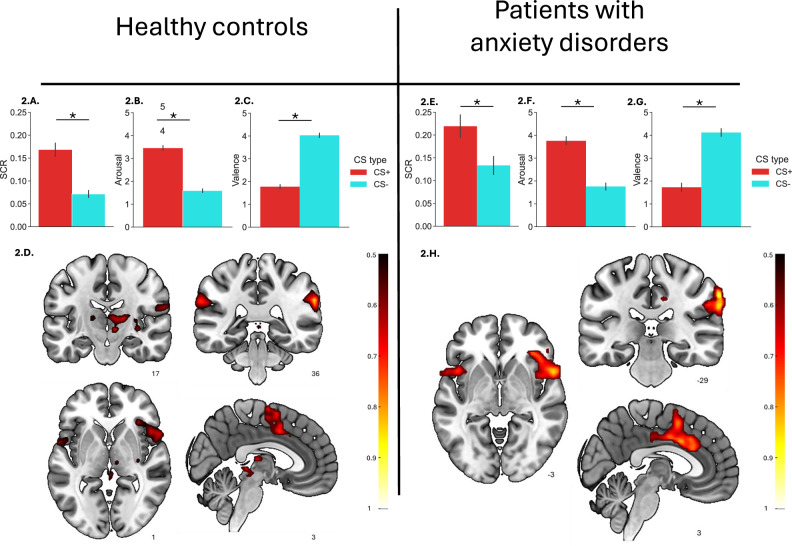
Delay fear conditioning in healthy controls (n = 102) and patients with anxiety disorders (n = 34). LEFT: Skin conductance responses (SCR) (**A**) subjective ratings of arousal (**B**) and valence (**C**) and functional magnetic resonance imaging (fMRI) responses (**D**) during delay fear conditioning in healthy controls. RIGHT: SCR (**E**) subjective ratings of arousal (**F**) and valence (**G**) and fMRI responses (**H**) during delay conditioning in patients with anxiety disorders. For subjective ratings, data refer to the responses to the CS+ or CS- at the end of the conditioning phase. For SCR, data refer to the average responses to the unreinforced CS+ trials and the CS- trials during conditioning. For fMRI, data refer to the CS + > CS- contrast, using the same trials as for the SCR. To facilitate visual comparison, the t-maps were converted to effect sizes by dividing them by the square root of the sample size. These maps were then thresholded at 0.5, representing the lower boundary of effect sizes within significant regions observed in the control group. Error bars indicate standard error of the mean (SEM). *p < *0.001*. fMRI figures display slices in the three orthogonal directions that best represent each group’s results. These images are not exhaustive, and full details can be found in the referenced supplementary tables. While slice selection may vary between control and patient groups, it is aimed at highlighting the most characteristic neural activations for each group across the studied contrasts.

In the aggregated analyses that included all trials, both controls (Fig. 2A, B, C) and patients (Fig. 2E, F, G) showed evidence of successful delay fear conditioning in SCR and subjective ratings, with significantly larger SCR to the CS+ compared to the CS-, and significantly higher arousal and lower valence ratings for the CS+ compared to the CS- (all *ps* < 0.001). Although SCR was, on average, higher among patients than controls (F(1,127) = 6.20, *p* < 0.05, η^2^_p_ = 0.047), the Group × CS type interaction was not significant for SCR, arousal, or valence (all F*s* ≤ 0.33, all *ps* ≥ 0.56; Supplementary Table 4), indicating no significant between-group differences in differential conditioning (CS+ vs. CS − ). However, our planned analyses focused on safety learning showed that SCR to the CS− was greater in patients compared with controls [Patients: M(SD) = 0.13 (0.11); Controls: M(SD) = 0.07 (0.08); t(127) = − 3.39, *p* < 0.001]. Notably, the absence of a significant Group × CS type interaction also indicates that SCR to the CS+ was elevated in patients relative to controls. Similar effects were not observed for arousal or valence ratings.

**Fig. 3:**
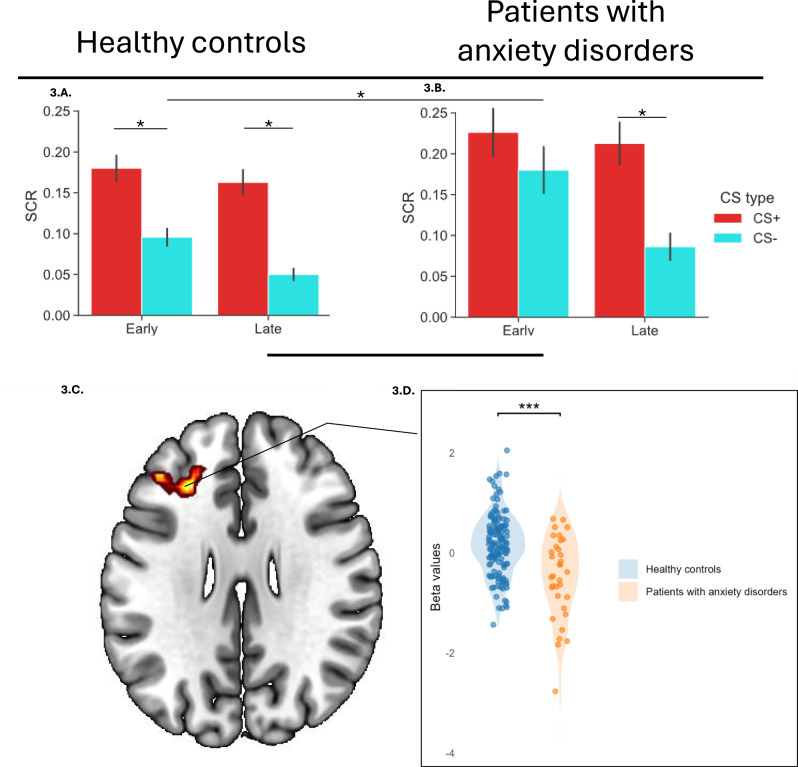
Delay fear conditioning responses during early and late phases in healthy controls (n = 102) and patients with anxiety disorders (n = 34). Skin conductance responses (SCR) data represent average responses to the first and last five CS+ and CS− trials (unreinforced CS+ trials only) (**A**, **B**). fMRI results for the CS − > fixation cross contrast (safety learning) during the same trials show significantly less activation in patients compared to healthy controls during early conditioning within a cluster in the left dorsolateral prefrontal cortex (**C**). Violin plot depicting mean beta values within the significant cluster for each group (**D**). *p < 0.05, **p < 0.001.

In the disaggregated (early and late) SCR analyses, the ANOVA revealed a significant main effect of group (F(1,127) = 6.20, *p* = 0.014, η²*p* = 0.047) and a three-way interaction between CS type, group, and phase (F(1,127) = 4.18, *p* = 0.043, η²*p* = 0.032; Supplementary Table 5). Post-hoc analyses revealed that controls exhibited successful differential conditioning (CS+ vs CS-) during early conditioning (*p* < 0.05; Fig. 3A), whereas patients did not (*p* = 0.974; Fig. 3B). This reflected the fact that patients exhibited higher SCR to the CS− than controls during early conditioning [Patients:*M* (SD):0.18 (0.16); Controls: *M*(SD): 0.09 (0.10), (t(127) = − 3.45, *p* < 0.001)] (Fig. 3A, B).

The aggregated fMRI analyses provided evidence of successful conditioning in controls (Fig. 2D) and patients (Fig. 2H). Specifically, the CS + > CS− contrast revealed increased activation in regions previously associated with fear conditioning, [8, 9] including the supramarginal gyrus, anterior insular cortices (extending into the frontal operculum), anterior and middle cingulate cortex, and thalamus (see Supplementary Tables 6 and 7). Group differences were negligible in the aggregated and disaggregated analyses for the CS+ vs CS- contrast.

For the CS– > Cfix contrast (safety learning), the aggregated fMRI analyses revealed no group differences. However, in the disaggregated analyses, patients showed significantly reduced activation to the safety cue (CS–) in the left dorsolateral prefrontal cortex (dlPFC) during the early phase of conditioning (Fig. 3C, D). No group differences were observed in response to the CS– during the late phase.

### Fear reversal

**Fig. 4:**
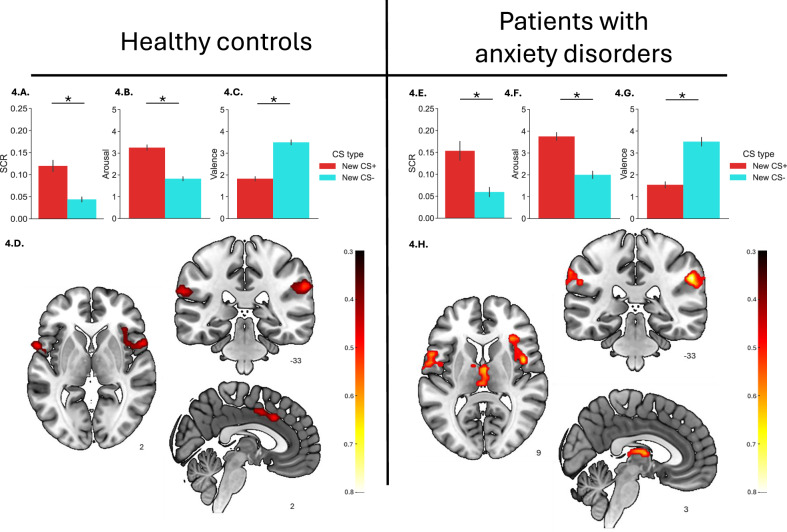
Fear reversal in healthy controls (n = 102) and patients with anxiety disorders (n = 34). LEFT: SCR (**A**) subjective ratings of arousal (**B**) and valence (**C**) and fMRI responses (**D**) during fear reversal in healthy controls. RIGHT: SCR (**E**) subjective ratings of arousal (**F**) and valence (**G**) and fMRI responses (**H**) during fear reversal in patients with anxiety disorders. For subjective ratings, data refer to the responses to the new CS+ or new CS- after fear reversal. For SCR, data refer to the average responses to the unreinforced new CS+ trials and the new CS- trials during fear reversal. For fMRI, data refer to the new CS + > new CS- contrast using the same trials as for SCR. To facilitate visual comparison, the t-maps were converted to effect sizes by dividing them by the square root of the sample size. These maps were then thresholded at 0.5, representing the lower boundary of effect sizes within significant regions observed in the control group. Error bars indicate standard error of the mean (SEM). *p < *0.001*. fMRI figures display slices in the three orthogonal directions that best represent each group’s results. These images are not exhaustive, and full details can be found in the referenced supplementary tables. While slice selection may vary between control and patient groups, it is aimed at highlighting the most characteristic neural activations for each group across the studied contrasts.

For the aggregated analyses, both controls (Fig. 4A, B, C) and patients (Fig. 4E, F, G) showed evidence of successful fear reversal in SCR and subjective ratings, with significantly larger SCR to the new CS+ compared to the new CS-, and significantly higher arousal and lower valence ratings scores to the new CS+ compared to the new CS- (all Fs ≥ 87.19, all *ps* < 0.001). The Group × CS type interaction was not significant for SCR, arousal, or valence (all Fs ≤ 2.22, all *ps* ≥ 0.138) (Supplementary Table 8), indicating to significant group differences. Similar conclusions were evident for the disaggregated SCR analyses (Supplementary Tables 9).

fMRI results (new CS + >new CS-contrast) showed evidence of successful fear reversal in both controls (Fig. 4D) and patients (Fig. 4H), with increased brain activation across several brain regions, including the supramarginal gyrus, anterior insula (extending to the temporal pole), thalamus, and midcingulate cortex (see Supplementary Tables 10 and 11**)**. Note that the regions activated during reversal are largely overlapping to those observed during conditioning. Group differences were negligible in the aggregated and disaggregated analyses.

Rather than calculating fear reversal by directly comparing CS+ and CS− responses during reversal, some recent fear reversal studies have separately assessed *threat reversal* and *safety reversal*. [27] In principle, this approach provides a more precise measure of the ability to update and inhibit conditioned fear responses as stimulus-outcome contingencies change. Nevertheless, groups did not differ in SCR, subjective ratings, or neural activation during the switch from CS− to CS+ or vice versa (SCR/Ratings: all ts ≤ |1.47 | , all ps ≥ 0.145; fMRI: see Sup. Mat.**: Additional Analyses** and Supplementary Table 12).

### Trace fear conditioning

**Fig. 5:**
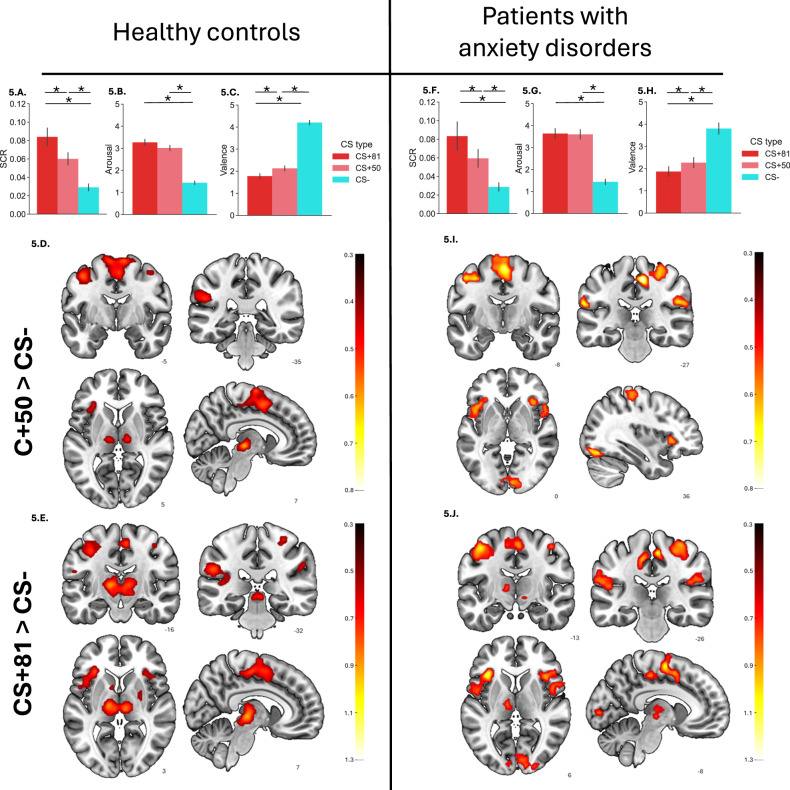
Trace fear conditioning in healthy controls (n = 102) and patients with anxiety disorders (n = 34). LEFT: SCR (**A**) subjective ratings of arousal (**B**) and valence (**C**) and fMRI responses for the contrasts CS + 50 > CS- (**D**) and CS + 81 > CS- (**E**) during trace fear conditioning in healthy controls. RIGHT: SCR (**F**) subjective ratings of arousal (**G**) and valence (**H**) and fMRI responses for the contrasts CS + 50 > CS- (**I**) and CS + 81 > CS- (**J**) during trace fear conditioning in patients with anxiety disorders. For subjective ratings, data refer to the responses to the CS + 50, CS + 81 or CS- at the end of the trace conditioning phase. For SCR, data refer to the average responses to the unreinforced CS + 50 and CS + 81 trials and the CS- trials during trace conditioning. For fMRI, data refer to the above-mentioned contrasts using the same trials as for SCR. To facilitate visual comparison, the t-maps were converted to effect sizes by dividing them by the square root of the sample size. These maps were then thresholded at 0.5, representing the lower boundary of effect sizes within significant regions observed in the control group. Error bars indicate standard error of the mean (SEM). *p < *0.001*. fMRI figures display slices in the three orthogonal directions that best represent each group’s results. These images are not exhaustive, and full details can be found in the referenced supplementary tables. While slice selection may vary between control and patient groups, it is aimed at highlighting the most characteristic neural activations for each group across the studied contrasts.

There was evidence of successful trace conditioning within both groups and most measures. Both controls (Fig. 5A, B, C) and patients (Fig. 5F, G, H) exhibited significantly larger SCR, higher arousal, and lower valence to CS + 81 and CS + 50 compared to CS− (all *ps* < 0.001). When comparing CS + 81 to CS + 50, both controls and patients showed significantly larger SCRs (Fig. 5A, F) and lower valence ratings (Fig. 5C, H**)** for CS + 81 than CS + 50 (*ps* < 0.02). In contrast, arousal ratings to CS + 81 and CS + 50 did not differ significantly in either the controls (Fig. 5B) or the patients (Fig. 5G) (*ps* > 0.99). The Group × CS type interaction was not significant for SCR, arousal, or valence (all Fs(2,244) < 2.26, all *ps* > 0.11) (Supplementary Table 13). The main effect of group was also non-significant, indicating that SCR, arousal, and valence levels were generally similar across groups (all Fs(1,122) < 3.46, all *ps* > 0.07). Similar conclusions were evident for the disaggregated analyses (Supplementary Table 14).

fMRI findings provided evidence of successful trace conditioning in both controls (Fig. 5D, E) and patients (Fig. 5I, J) for the contrasts CS + 50 > CS− and CS + 81 > CS − . In each group, these contrasts were associated with increased activation across several regions, including the thalamus, supplementary motor area (SMA), supramarginal gyrus, precentral/postcentral gyri, and the insula extending into the inferior frontal operculum (see Supplementary Tables 15–18). For the CS + 81 > CS + 50 contrast, controls showed increased activation in several regions, including the temporal/occipital middle gyri, putamen, hippocampus, thalamus, and precentral/postcentral gyri (see Supplementary Table 19 and Supplementary Fig. 3). However, this contrast did not yield significant activation increases in patients.

Direct group comparisons revealed no significant differences in brain activation between patients and controls for any of the three contrasts (CS + 50 > CS − , CS + 81 > CS − , CS + 81 > CS + 50), indicating broadly similar neural responses. Comparable patterns were observed in the disaggregated analyses.

#### Sensitivity analyses

None of the key conclusions regarding patient–control differences changed when gender or age were included as covariates (see Supplementary Tables 20–22). When task order was included as a factor, a significant patient–control difference emerged in the disaggregated analyses for the CS + > CS− contrast. Specifically, during early delay fear conditioning, patients showed greater deactivation to the CS− in a cluster located in the left dlPFC (Supplementary Table 23). For all other measures and contrasts, task order did not materially alter the main findings regarding patient–control differences (Supplementary Tables 24–26).

## Discussion

This is the first study to jointly examine delay conditioning, fear reversal, and trace conditioning across subjective, psychophysiological, and neural measures in unmedicated individuals with anxiety disorders and matched controls. Overall, our findings suggest that individuals with anxiety disorders show impaired safety learning during the early stages of delay conditioning but do not exhibit marked alterations in either fear reversal or in trace conditioning.

Previous studies using delay fear conditioning paradigms in unmedicated individuals with anxiety disorders, as currently defined, remain limited. Pöhlchen et al. [31] found no significant differences in subjective (expectancy ratings) or psychophysiological (SCR, FPS, pupillometry) measures of conditioned fear (CS+ vs CS- difference) when comparing patients with anxiety disorders (specific phobia, SAD, agoraphobia, and PD) to healthy controls. Similarly, several prior studies focusing on GAD, SAD and PD patients reported no differences between patients and controls in psychophysiological (FPS or SCR) or subjective (expectancy ratings) conditioned fear measures. [32–36]

In our aggregated analysis, which included all trials, we also found no group differences in cue differentiation (CS+ vs. CS-) during delay conditioning. However, patients showed heightened SCR responses to the CSs, showing overall higher physiological responding. Specifically, patients showed increased safety cue (CS-) SCR. Although this effect was evident in our aggregated results, phase-specific analyses indicated that this alteration largely is most pronounced during the early phase of delay fear conditioning. Mirroring this effect, fMRI results revealed decreased activation to the safety cue (CS-) in the left dlPFC during the early phase of conditioning. The dlPFC plays a key role in emotion regulation, [37–39] and greater dlPFC activation in response to safety cues has been linked to fear inhibition. [40, 41] Thus, reduced dlPFC engagement in patients may reflect difficulties in downregulating responses to the CS − , particularly during the early stages of learning Pavlovian safety associations. Methodologically, our observations underscore the importance of cue- and phase-specific analyses for understanding the alterations in fear learning that mark individuals with pathological anxiety. [42]

Contrary to our expectations, we did not find differences in fear reversal between our patient and control groups. Our results are aligned with those of Savage et al. [43], who also found no differences in subjective ratings, SCR, or brain activation during fear reversal in unmedicated patients with SAD compared to healthy controls or patients with major depressive disorder. Roberts et al. [15] reported reduced differential SCR responses in GAD patients compared to controls during the early but not the late phase of fear reversal. This study included mostly (79%) medicated participants, and as noted in the introduction, medication may be a key confound in fear learning studies. [16] Variation in the type of CSs used could also influence these differences: unlike Savage et al. and the current study, which employed geometric figures, Roberts et al. [15] used angry faces as CS. Two additional factors may help explain the absence of group differences for fear reversal. First, fMRI reversal effects are typically modest and often restricted to specific regions of interest (e.g., OFC, vmPFC); therefore, our conservative whole-brain corrections may have obscured potential group differences. Second, from a theoretical perspective, reversal learning deficits may be more characteristic of other mental disorders, such as OCD, than of anxiety disorders. [15, 44]

We anticipated that using a trace conditioning paradigm, a “weak situation” (see **Introduction**), with two CS+ stimuli featuring different pairing rates would enhance the detection of fear learning differences between individuals with anxiety disorders and controls. However, our findings did not support this hypothesis. To our knowledge, ours is the first study that has directly compared trace fear conditioning between patients with anxiety disorders and healthy controls. Our trace interval was relatively short (1.6–2 s), and it is possible that differences may have emerged with longer intervals—a possibility that warrants further exploration.

Overall, and in line with several previous reports, [31–36, 43] our results suggest that anxiety disorders, as a group, are not characterized by robust alterations in most fear learning processes investigated here. However, it remains possible that diagnoses not represented here (e.g., panic disorder) do show such alterations. Additionally, disruptions in other fear learning mechanisms—such as fear extinction learning, [45] fear generalization [46] or fear extinction recall [30], may characterize anxiety disorders. The “anxiety disorders” category has changed over the years and the current versions of the most employed classification systems (DSM-5 [17] and ICD-11 [47]) do not include post-traumatic stress disorder (PTSD). A recent large-scale study on the neural correlates of delay fear conditioning [9] found increased brain activation during fear conditioning in multiple regions among patients with “anxiety-related disorders” (a category including anxiety disorders, OCD, and PTSD) compared to healthy controls. Using linear models and normative modeling analyses, [48] the study further revealed that alterations in delay fear conditioning were characteristic of PTSD and OCD but not of GAD or SAD. When considered alongside our findings and previous research on PTSD, [49] these results suggest that fear conditioning abnormalities, at least in the context of delay fear conditioning, may be more strongly associated with PTSD than with other “anxiety-related disorders.” Given that PTSD and OCD are often linked to greater severity and functional impairment compared to other anxiety disorders, [50, 51] and that fear conditioning abnormalities have also been reported in a broader spectrum of mental disorders [52] (e.g., schizophrenia), we speculate that such abnormalities may serve as a transdiagnostic marker of severity rather than being specific to any single diagnosis. In this view, altered fear conditioning could reflect a general liability dimension that varies continuously with symptom burden, rather than mapping into specific psychiatric categorization. This hypothesis could be tested in future research by incorporating fear conditioning measures across individuals with various mental disorder diagnoses and assessing them using a standardized measure of severity and functional impairment.

Our null findings regarding differences between patients and controls for most fear learning contrasts may also reflect methodological factors. Human fear conditioning experiments are influenced by multiple variables, including the type and number of CSs and USs, the reinforcement (pairing) rate, and the measures used to assess fear responses and how they are collected, yet the effects of these factors are only beginning to be systematically understood. [9, 19] For example, some authors have emphasized the importance of using fear-relevant CSs (e.g., angry faces in SAD) when studying patients with anxiety or fear-related disorders. [53] Different reinforcement schedules can also affect the magnitude and pattern of conditioned responses. [14] Finally, and particularly relevant for our study, the (f)MRI environment itself is stressful, which can alter both neural and behavioral responses. This stress may increase variability in the control group, potentially reducing statistical power to detect differences between patients and controls. [7, 54, 55]

Finally, it is also noteworthy that previous research suggests that fear-conditioning paradigms rely on partly distinct neural and cognitive mechanisms. Delay conditioning -specially in the rodent literature- primarily reflects amygdala-based associative learning. [56] Reversal learning additionally engages prefrontal and striatal circuits that support cognitive flexibility. [57] Finally, trace conditioning depends on hippocampal and working-memory processes to bridge the CS–US interval. [10] It is therefore plausible that anxiety-related alterations are more pronounced in basic associative learning, as observed in delay conditioning, whereas group differences in reversal or trace conditioning are less robust. [58]

Our study has several strengths and limitations. A key strength is our well-characterized patient sample, consisting of non-medicated adult individuals with a primary diagnosis of an anxiety disorder based on current classification systems and little-to-no comorbidity. These individuals were thoroughly phenotyped, exhibiting significantly higher anxiety symptom scores than controls across all psychometric measures. However, our sample was not entirely homogeneous, as it did not consist solely of patients with a single anxiety disorder (e.g. only GAD or SAD). Although GAD is currently classified as an anxiety disorder, it is often conceptualized not as a prototypical ‘fear disorder’ (like SAD) but rather as a ‘misery disorder,’ due to its strong associations with chronic negative affect and depression. [59] Additionally, some anxiety disorders (e.g., specific phobia, PD) were not represented. However, the prevailing assumption in the field is that fear learning alterations are a common feature across *all* anxiety disorders. [14, 46] Although our sample size was relatively small, the three paradigms examined—delay fear conditioning, fear reversal, and trace fear conditioning—elicited robust fear responses at the subjective, psychophysiological, and neural levels *within each group*. This indicates sufficient assay sensitivity, except for certain measures in the CS + 81 vs. CS + 50 contrast in trace conditioning. Moreover, for each process, we included multiple operationalizations—such as all trials, early and late phases, and an alternative approach to fear reversal. However, there are numerous other possible ways to operationalize fear learning processes. [60] We were also unable to obtain valence and arousal ratings specifically for early versus late trials, as subjective ratings were collected only at the end of the task. Finally, another limitation concerns the interpretation of SCRs on non-reinforced trials. SCRs were quantified in a CS-locked time window on trials in which the unconditioned stimulus (UCS) was omitted, following common practice in human fear-conditioning research. However, classical psychophysiological work has shown that SCRs on non-reinforced trials may reflect not only conditioned responding to the CS, but also responses related to the omission of the expected UCS (i.e., third-interval responses). [61, 62] Accordingly, SCRs in the present study should be interpreted as an index of differential autonomic responding to CS+ versus CS − , rather than as a pure measure of conditioned responding in the strict psychophysiological sense. Future studies using interval-specific SCR modeling may help to further dissociate anticipatory and omission-related components of autonomic responses.

In summary, we did not find robust evidence that individuals with anxiety disorders (GAD and SAD) exhibit significant alterations in delay or trace fear conditioning or fear reversal, but they may be characterized by impaired safety learning. It is possible that other fear learning processes better characterize these disorders, or that such abnormalities are more relevant to other mental disorders. Future research should explore whether fear-learning abnormalities are more indicative of disorder severity rather than diagnostic status.

## Supplementary information

Supplementary material

## Data Availability

The data that support the findings of this study are available from the corresponding author, upon reasonable request.

## Code availability

The code that supports the findings of this study is available from the corresponding author upon reasonable request.
