# Supplementary material for: Fear learning in unmedicated patients with anxiety disorders: a comparison of delay conditioning, fear reversal, and trace conditioning
Source: Transl Psychiatry. 2026 Apr 2;16:274. doi: 10.1038/s41398-026-03996-6 (PMC13184327; doi:10.1038/s41398-026-03996-6)
Supplement: Supplementary file 1 — Supplementary material [file 41398_2026_3996_MOESM1_ESM.docx]

***Fear learning in unmedicated patients with anxiety disorders: a comparison of delay conditioning, fear reversal, and trace conditioning.***

Supplementary Material

|  | **Page** |
| --- | --- |
| **Supplementary Methods** |  |
| **Recruitment procedures** | 2 |
| **Self-report measures** | 2 |
| **Fear learning assessment**  Instructions  Unconditioned stimuli  Conditioned stimuli | 3 |
| **Measures of conditioned fear**  Subjective ratings  Skin conductance responses  Data acquisition  Data processing and misingness  Brain activation  Imaging acquisition  Anatomical data (pre)processing  Functional data (pre)preprocessing | 3 |
| **Pre-conditioning analyses** | 5 |
| **Additional analyses**  Early and late  Fear reversal – threat and safety reversal | 5 |
| **Supplementary Tables** | 7 |
| **Supplementary Figures** | 33 |
| **Supplementary References** | 36 |
|  |  |

**SUPPLEMENTARY METHODS**

**Recruitment procedures**

We screened a large number (n = 840) of adult individuals (age ≥ 18) from the university community, including students and staff, using the Spanish version ^1^ of the State-Trait Anxiety Inventory–Trait (STAI-T) subscale ^2^ via a secure web system. We aimed to recruit participants with different levels of trait anxiety (including individuals with a current anxiety disorder). Therefore, STAI-T data were stratified into quartiles, and individuals who met preliminary inclusion criteria were selected from each stratum. These individuals (n = 361) were assessed during a telephone interview by an experienced clinician who administered the Spanish version ^3^ of the Mini International Neuropsychiatric Interview ^4^ and confirmed that potential participants fulfilled the inclusion/exclusion criteria. Inclusion criteria were: 1) age between 18 and 36 years, 2) owning a smartphone (because the larger study included smartphone-based assessments), and 3) being willing to participate in a neuroimaging assessment. Exclusion criteria were 1) current or previous severe medical disorder or current medication that could interfere with the study objectives (as per self-report), 2) current or past mental disorder (except current anxiety disorder, see below), or 3) current substance use (except occasional use of alcohol and other recreational drugs, or tobacco), as per the MINI, and 4) any contraindication to neuroimaging assessment. Those who met the inclusion/exclusion criteria (n = 206) gave written informed consent and participated in the laboratory session reported in this manuscript. Twenty-seven participants were excluded from MRI analysis (5 because of incidental findings, and 22 due to motion artifacts or poor image quality), leaving 179 potential participants. Thirty-four of these 179 participants were given a diagnosis of a current anxiety disorder and made up the patient group. Among the rest (n = 135), and to enhance statistical power, we selected 3 controls for each patient, ensuring that the samples had a matched gender distribution (55.9% female) and no statistically significant differences in age (mean age 25.6 years in both, *p* = 1). These participants (n = 102) made up the control group.

For the fMRI analyses of the trace conditioning task, three patients and nine controls were excluded due to poor data quality. Additionally, for the SCR analyses, one patient and four controls were excluded from the delay/reversal fear-conditioning task due to recording artifacts, and four patients and eight controls were excluded from the trace conditioning task for the same reason. The final number of participants included in each analysis/task is shown in **Sup. Table 1.**

**Self-report measures**

Participants completed the Spanish versions of the following measures:

-Trait subscale of the *State-Trait Anxiety Inventory* (STAI-T) a 20-item questionnaire assessing trait anxiety (dispositional negative affect). ^1,2^ Total scores range from 0 to 60.

-*Intolerance of Uncertainty Scale* (IUS), a 27-item questionnaire assessing the tendency to react negativelyto uncertain situations. ^5,6^Total scores range from 0 to 135.

*-Liebowitz Social Anxiety Scale (LSAS),* a 24-item questionnaire assessing anxiety and avoidance of social situations.^7,8^ Total scores range from 0 to 144.

*-Screening scale for DSM-IV Generalized Anxiety Disorder*, a 12-item questionnaire assessing Generalized Anxiety Disorder symptoms.^9,10^ Total scores range from 0 to 12.

*-Penn State Worry Questionnaire-11* (PSWQ-11) a 11-item questionnaire assessing trait worry.^11,12^ Total scores range from 11 to 55.

*-Depression, Anxiety and Stress Scales* (DASS-21) a 21-item scale assessing depression, anxiety, and stress symptoms.^13,14^ Total scores range from 0 to 21 for each subscale.

**Fear learning assessment**

**Instructions**

Before starting the tasks (outside the scanner), participants were informed that they would, at some point during the session, view geometrical figures and experience electric shocks. They were also familiarized with the system used for recording subjective ratings.

**Unconditioned stimuli**

The two unconditioned stimuli (USs) were brief electric shocks, delivered as a quadratic pulse for the delay acquisition/reversal task and a sinusoidal pulse for the trace task. Shocks were administered via two MRI-compatible Ag/AgCl electrodes filled with electrolyte gel and delivered using a Biopac STMISOLA stimulator. Electrodes were placed on the left hand for the delay acquisition/reversal task and on the left forearm for the trace task. Delivering shocks to different locations helped minimize carryover or generalization effects between tasks. The intensity of both USs was individually calibrated inside the scanner using a staircase procedure to ensure the shocks were unpleasant but not painful (rated >7 on a 1–10 aversiveness scale, where 10 was maximum aversiveness). The procedure began at 30V, increasing in 10V increments until the participant indicated their maximum level of discomfort or the 100V maximum was reached.

**Measures of conditioned fear**

**Subjective ratings**

For the valence ratings, participants responded to the question "How unpleasant/pleasant did you find the [colour of the CS] sphere?”. Responses ranged from 1='very unpleasant' to 5='very pleasant'. For anxious arousal ratings, participants responded to the question: "How anxious did the [colour of the CS] sphere make you feel?". Responses ranged from 1='not anxious' to 5='very anxious'. Participants’ ratings were recorded using an MRI-compatible, three-button fiber-optic response box (Lumina 3G Controller, Cedrus Corporation), with which participants were familiarized prior to scanning

**Skin conductance responses**

*Data acquisition.* Skin conductance data were continuously acquired during both fear conditioning tasks using a Biopac EDA100c module, MP150 Amplifier, and AcqKnowledge 4.4.0 software (250-Hz sampling, 5 µSiemens/Volt gain, 10-Hz low-pass, DC high-pass). Skin conductance was collected from the volar surfaces of the distal phalanges of the third and fourth fingers of the left hand, using two Ag-AgCl, nonpolarizable electrodes and isotonic gel (GEL101).

*Data processing and missingness.* Data preprocessing was implemented using AcqKnowledge. Data were down-sampled (62.5 Hz) and smoothed to mitigate movement artifacts (63-sample median and 1-Hz low-pass filters). All SCRs were visually inspected. Trial-by-trial SCRs were quantified using custom-made MATLAB scripts as trough-to-peak responses with an onset latency of 1-6 s in the delay/reversal task and 1-5.3 s in the trace task. Trials with increases <0.02 µS or that lasted <0.5 s were scored as non-responses and set to a value of 0 µS. Trials with artifacts or excessive baseline activity were treated as missing responses. Using SCR raw data, participants showing non-valid responses in ≥ 75% of the CS trials followed by a US during delay conditioning or trace conditioning were classified as physiological non-responders and all SCR trials treated as missing responses. After excluding physiological non-responders, SCR amplitudes were normalized and range corrected separately for each task using the formula ln(1+ SCR)/ln(1 + MAX), where MAX was the individual’s maximum response to the US.

**Brain activation**

*Imaging Acquisition.* T1-weighted (T1w) anatomical scans were acquired using a three-dimensional fast-spoiled gradient, inversion-recovery sequence (TR=10.43 ms; TE=4.8 ms; flip=8°; slice thickness=0.75 mm; in-plane=0.75 × 0.75 mm; matrix=320 × 320; field-of-view=240 × 240). Functional data were acquired using a single-shot gradient-echo echo-planar imaging (EPI) sequence (TR=2,000 ms; TE=25 ms; flip=90°; slice thickness=3 mm; in-plane resolution=3 × 3 mm; matrix=80 × 80). Images were collected in the AC-PC plane to minimize potential susceptibility artifacts. For the delay fear conditioning task, one run comprising 480 volumes was acquired (total acquisition time: 15m 25s). For the trace conditioning task, one run of 458 volumes was collected (total acquisition time: 14m 13s)

*Anatomical data (pre)processing.* T1w images were corrected for intensity non-uniformity using the ANTs (version 2.2.0,6) N4BiasFieldCorrection algorithm,^15^ skull-stripped using a Nipype implementation of antsBrainExtraction.sh and the OASIS30ANTs as a target template, and segmented using the FSL (version 5.0.9 7) ^16^ fast algorithm. Brain surfaces were reconstructed using the FreeSurfer (version 6.0.1 8) ^17^ recon-all algorithm. Brain masks were refined using a variant of a previously described method for reconciling ANTS and FreeSurfer gray matter (GM) segments. ^18^ Volume-based spatial normalization to two standard brain-extracted templates (MNI152NLin2009cAsym and MNI152NLin6Asym) ^19^ was performed using the ANTs diffeomorphic algorithm.

*Functional data (pre)processing.* fMRI data were preprocessed using fMRIPrep 1.4.1,^20^ which is based on Nipype 1.2.0.^21^ Preprocessing included skull stripping, susceptibility distortion correction using field maps, and co-registration of each BOLD reference image to the participant's T1-weighted anatomical scan using boundary-based registration (bbregister, FreeSurfer).^22^ Slice timing correction was applied using AFNI’s 3dTshift, ^23^ and motion correction was performed with FSL’s mcflirt.^24^ All transformations (motion correction, distortion correction, co-registration, and spatial normalization) were combined and applied in a single resampling step using ANTs with Lanczos interpolation.^25^ The resulting BOLD time-series were normalized to MNI152NLin2009cAsym space and spatially smoothed with a 6 mm FWHM Gaussian kernel. For denoising, ICA-AROMA was used to identify and remove motion-related components;^26^ the analyses reported here used the non-aggressively denoised time-series. Nuisance regressors included six motion parameters, their temporal derivatives and quadratic terms, as well as anatomical and temporal CompCor components.^27^ These were computed after high-pass filtering (128 s cutoff), and the number of retained components was set to explain at least 50% of variance within the respective noise masks (white matter, CSF, or combined). Framewise displacement (FD) and DVARS were computed for each run. Volumes exceeding 0.5 mm FD or 1.5 standardized DVARS were flagged as motion outliers and excluded from first-level analyses.^28^ Unless otherwise stated, all analyses were conducted in MNI space using the 6 mm smoothed, non-aggressively denoised BOLD time-series.

**Pre-conditioning analysis**

Differences between responses to each to-be CS within each group were assessed using a paired t-test for the delay/reversal task (CS+ and CS-), and one-way repeated measures ANOVAs for the trace task (CS-, CS+50, and CS+81). Specifically, we compared the last CS trial during preconditioning for SCR and brain activation, as well as arousal and valence ratings collected after the preconditioning phase.

**Additional Analyses**

Fear reversal – threat and safety reversal

For subjective ratings, we defined threat reversal as the difference between arousal or valence ratings for the CS+ during reversal ("new CS+") and the CS− during conditioning. Safety reversal was defined as the difference between arousal or valence ratings for the CS− during reversal ("new CS−") and the CS+ during conditioning. For SCR, threat reversal was operationalized as the sum of all SCR responses to the unreinforced new CS+ (CS+ during reversal, 10 trials) minus the sum of responses to the CS− during conditioning. Similarly, safety reversal was defined as the sum of all SCR responses to the new CS− (CS− during reversal) minus the sum of responses to unreinforced CS+ during conditioning (10 trials). For fMRI analyses, first-level contrast images were generated by comparing new CS+ vs. CS− for threat reversal and new CS− vs. CS+ for safety reversal. Both contrasts used the same trials as the SCR analysis. Threat and safety reversal for subjective ratings and SCR were analyzed separately using two sample t-tests to compare controls and patients. fMRI group differences for each contrast were assessed using two-sample t-tests.

**SUPPLEMENTARY TABLES**

**Supplementary Table 1.** Number of participants included in each analysis

|  | Delay /Reversal task | | Trace task | |
| --- | --- | --- | --- | --- |
|  | Healthy controls | Patients | Healthy controls | Patients |
| fMRI analysis | 102 | 34 | 93 | 31 |
| SCR analysis | 98 | 33 | 94 | 30 |

**Supplementary Table 2.** Paired t-test results for the effects of CS type on SCR, arousal, and valence during preconditioning of the delay fear-conditioning task.

| Effect | t-test | *t* | *df* | *p* |
| --- | --- | --- | --- | --- |
|  | SCR | 0.44 | 95 | .661 |
| Healthy Controls | Arousal ratings | 0.58 | 95 | .566 |
|  | Valence ratings | 0.65 | 95 | .517 |
|  | SCR | -0.89 | 32 | .377 |
| Patients | Arousal ratings | 0.24 | 32 | .812 |
|  | Valence ratings | -0.37 | 32 | .712 |

**Supplementary Table 3.** ANOVAs results for the effects CS type on SCR, arousal, and valence during preconditioning of the trace fear-conditioning task.

| Group | ANOVA | *F* | *df* | *p* | η^2^ |
| --- | --- | --- | --- | --- | --- |
|  | SCR | 7.06 | 2, 186 | .001 | <.05 |
| Healthy Controls | Arousal ratings | 0.48 | 2, 186 | .606 | <.01 |
|  | Valence ratings | 1.72 | 2, 186 | .182 | <.01 |
|  | SCR | 0.77 | 2, 58 | .467 | <.01 |
| Patients | Arousal ratings | 1.39 | 2, 58 | .163 | <.05 |
|  | Valence ratings | 1.87 | 2, 58 | .256 | <.05 |

**Supplementary Table 4.** ANOVAs results for the effects of group and CS type on SCR, arousal, and valence during delay conditioning

| ANOVA | Effect | *F* | *df* | *p* | η^2^ |
| --- | --- | --- | --- | --- | --- |
|  | G | 6.20 | 1, 127 | .014 | .047 |
| SCR | CS | 72.64 | 1, 127 | <.001 | .364 |
|  | CS x G | 0.33 | 1, 127 | .566 | .003 |
|  | G | 1.91 | 1, 127 | .169 | .015 |
| Arousal ratings | CS | 278.53 | 1, 127 | <.001 | .687 |
|  | CS x G | 0.34 | 1, 127 | .560 | .003 |
|  | G | .004 | 1, 127 | .837 | <.001 |
| Valence ratings | CS | 273.56 | 1, 127 | <.001 | .683 |
|  | CS x G | 0.26 | 1, 127 | .609 | .002 |

*Note*: G = Group; CS = CS type

**Supplementary Table 5.** ANOVA results for the effects of group, CS type, and phase (early and late) on SCR during delay fear conditioning.

| Effect | *F* | *df* | *p* | η^2^ |
| --- | --- | --- | --- | --- |
| G | 6.20 | 1, 127 | .014 | .047 |
| P | 28.22 | 1, 127 | <.001 | .182 |
| P x G | 1.92 | 1, 127 | .168 | .015 |
| CS | 72.64 | 1, 127 | <.001 | .364 |
| CS x G | 0.33 | 1, 127 | .566 | .003 |
| P x CS | 18.18 | 1, 127 | <.001 | .125 |
| P x CS x G | 4.18 | 1, 127 | .043 | .032 |

*Note*: G = Group, P = Phase, CS = CS type

**Supplementary Table 6.** Brain activations during delay fear conditioning (CS+>CS-) for healthy controls.

*Note:* AAL = Automated Anatomical Labeling, FWER = Family Wise Error Rate, N = Number, MNI = Montreal Neurological Institute

| AAL Region | Cluster *p* value - FWER | N voxels | *t* | MNI (x,y,z) | Peak *p* value  uncorrected |
| --- | --- | --- | --- | --- | --- |
| SupraMarginal_R | <0.001 | 255 | 8.85 | 63, -36, 30 | <0.001 |
|  |  |  | 7.38 | 54, -30, 30 | <0.001 |
|  |  |  | 6.82 | 63, -21, 21 | <0.001 |
| SupraMarginal_L | <0.001 | 162 | 7.96 | -63, -39, 30 | <0.001 |
|  |  |  | 6.46 | -66, -24, 27 | <0.001 |
|  |  |  | 5.99 | -60, -24, 21 | <0.001 |
| Insula_R | <0.001 | 189 | 7.55 | 48, 18, -3 | <0.001 |
|  |  |  | 7.09 | 57, 15, 3 | <0.001 |
|  |  |  | 6.5 | 33, 27, 0 | <0.001 |
| Thalamus_R | <0.001 | 163 | 7.38 | 9, -18, -3 | <0.001 |
|  |  |  | 7.11 | 18, -18, 9 | <0.001 |
|  |  |  | 6.68 | 9, -18, 9 | <0.001 |
| Temporal_Sup_R | <0.001 | 18 | 7 | 48, -24, -3 | <0.001 |
| Cingulum_Mid | <0.001 | 182 | 6.79 | 0, 6, 42 | <0.001 |
|  |  |  | 6.75 | -6, 12, 39 | <0.001 |
|  |  |  | 6.5 | 3, 3, 60 | <0.001 |
| Rolandic_Oper_L | <0.001 | 62 | 6.77 | -57, 3, 3 | <0.001 |
|  |  |  | 6.49 | -51, 9, -3 | <0.001 |
| Insula_L |  |  | 5.45 | -40,19,-2 | <0.001 |
| Cerebelum_6_L | <0.001 | 19 | 6.32 | -30, -63, -21 | <0.001 |
| Thalamus_L | <0.001 | 33 | 6.31 | -15, -12, 9 | <0.001 |
|  |  |  | 5.76 | -6, -9, 9 | <0.001 |
| Cerebelum_6_R | <0.001 | 11 | 6.19 | 36, -57, -27 | <0.001 |
|  |  |  | 5.61 | 30, -63, -24 | <0.001 |

**Supplementary Table 7.** Brain activations during delay fear conditioning (CS+ vs. CS-) for patients with anxiety disorders.

| AAL Region | Cluster *p* value FWER | N voxels | *t* | MNI (x,y,z) | Peak *p* value uncorrected |
| --- | --- | --- | --- | --- | --- |
| SupraMarginal_R | <0.001 | 273 | 6.22 | 66, -39, 30 | <0.001 |
|  |  |  | 5.47 | 66, -30, 39 | <0.001 |
|  |  |  | 5.23 | 66, -27, 27 | <0.001 |
| Cingulum_Ant | <0.001 | 466 | 5.26 | -3, 24, 27 | <0.001 |
| Cingulum_Mid |  |  | 4.65 | 3, 9, 33 | <0.001 |
|  |  |  | 4.41 | 6, 9, 54 | <0.001 |
| Temporal_Pole_Sup_R | <0.001 | 269 | 4.91 | 57, 12, -3 | <0.001 |
|  |  |  | 4.29 | 39, 30, -6 | <0.001 |
| Insula_R |  |  | 4.26 | 45, 9, 0 | <0.001 |
| Insula_L | <0.01 | 171 | 4.35 | -45, 12, -6 | <0.001 |
|  |  |  | 4.1 | -36, 6, 6 | <0.001 |
|  |  |  | 3.76 | -54, 9, 12 | <0.001 |

*Note:* AAL = Automated Anatomical Labeling, FWER = Family Wise Error Rate, N = Number, MNI = Montreal Neurological Institute

**Supplementary Table 8.** ANOVAs results for the effects of group and CS type on SCR, arousal, and valence during fear reversal.

| ANOVA | Effect | *F* | *df* | *p* | η2 |
| --- | --- | --- | --- | --- | --- |
|  | G | 1.79 | 1, 127 | .184 | .014 |
| SCR | CS | 87.19 | 1, 127 | <.001 | .683 |
|  | CS x G | 0.80 | 1, 127 | .371 | .006 |
|  | G | 3.10 | 1, 127 | .081 | .024 |
| Arousal ratings | CS | 221.59 | 1, 127 | <.001 | .636 |
|  | CS x G | 2.22 | 1, 127 | .138 | .017 |
|  | G | 0.72 | 1, 127 | .395 | .006 |
| Valence ratings | CS | 165.94 | 1, 127 | <.001 | .566 |
|  | CS x G | 1.07 | 1, 127 | .303 | .008 |

*Note*: G = Group; CS = CS type

**Supplementary Table 9.** ANOVA results for the effects of group, CS type, and phase on SCR during fear reversal.

| Effect | *F* | *df* | *p* | η2 |
| --- | --- | --- | --- | --- |
| G | 1.79 | 1, 127 | .184 | .014 |
| P | 46.64 | 1, 127 | <.001 | .269 |
| P x G | 1.43 | 1, 127 | .233 | .011 |
| CS | 87.19 | 1, 127 | <.001 | .407 |
| CS x G | 0.80 | 1, 127 | .372 | .006 |
| P x CS | 0.45 | 1, 127 | .502 | .004 |
| P x CS x G | 0.01 | 1, 127 | .909 | .000 |

Note*: G = Group, P = Phase, CS = CS type*

**Supplementary Table 10.** Brain activations during fear reversal (new CS+ vs. new CS-) for healthy controls.

| AAL Region | Cluster *p* value FWER | N voxels | *t* | MNI (x,y,z) | Peak *p* value uncorrected |
| --- | --- | --- | --- | --- | --- |
| SupraMarginal_R | <0.001 | 116 | 7.27 | 60, -30, 30 | <0.001 |
| SupraMarginal_L | <0.001 | 60 | 6.33 | -66, -36, 24 | <0.001 |
|  |  |  | 4.78 | -63, -24, 24 | <0.001 |
| Temporal_Pole_Sup_L | <0.001 | 41 | 6.31 | -60, 9, 0 | <0.001 |
| Temporal_Pole_Sup_R | <0.001 | 146 | 6.07 | 57, 12, 0 | <0.001 |
| Insula_R |  |  | 6.03 | 36, 24, 6 | <0.001 |
|  |  |  | 5.91 | 36, 15, 6 | <0.001 |
| Cingulum_Mid | <0.001 | 70 | 6 | 3, 18, 36 | <0.001 |
|  |  |  | 5.66 | 0, 3, 42 | <0.001 |

*Note:* AAL = Automated Anatomical Labeling, FWER = Family Wise Error Rate, N = Number, MNI = Montreal Neurological Institute

**Supplementary Table 11.** Brain activations during fear reversal (new CS+ vs. new CS-) for patients with anxiety disorders.

| AAL Region | Cluster *p* value FWER | N voxels | *t* | MNI (x,y,z) | Peak *p* value uncorrected |
| --- | --- | --- | --- | --- | --- |
| SupraMarginal_R | <0.01 | 139 | 5.67 | 57, -33, 33 | <0.001 |
|  |  |  | 4.73 | 63, -30, 27 | <0.001 |
|  |  |  | 3.86 | 54, -21, 24 | <0.001 |
| SupraMarginal_L | <0.001 | 178 | 5.01 | -57, -42, 33 | <0.001 |
|  |  |  | 4.2 | -63, -39, 39 | <0.001 |
|  |  |  | 3.65 | -66, -27, 18 | <0.001 |
| Insula_R | <0.001 | 197 | 4.8 | 45, 3, 9 | <0.001 |
|  |  |  | 4.69 | 60, 9, -3 | <0.001 |
|  |  |  | 4.35 | 36, 27, 9 | <0.001 |
| Temporal_Pole_Sup_L | <0.001 | 184 | 4.72 | -60, 12, -6 | <0.001 |
|  |  |  | 4.25 | -60, 3, 3 | <0.001 |
|  |  |  | 4.22 | -57, 9, 9 | <0.001 |
| Thalamus | <0.05 | 93 | 4.39 | 3, -6, 9 | <0.001 |
|  |  |  | 4.22 | -3, -24, 6 | <0.001 |
|  |  |  | 3.5 | -12, -6, 12 | <0.001 |

*Note:* AAL = Automated Anatomical Labeling, FWER = Family Wise Error Rate, N = Number, MNI = Montreal Neurological Institute

**Supplementary Table 12**. T-test results on SCR, arousal and valence for the comparison of threat reversal (new CS+ vs. CS-) and safety reversal (new CS- vs. CS+) between healthy controls and patients with anxiety disorders.

|  | Measure | *t* | *df* | *p* | Cohen's *d* |
| --- | --- | --- | --- | --- | --- |
| Threat reversal | SCR | 1.429 | 127 | .155 | 0.2884 |
|  | Valence | 1.400 | 127 | .164 | 0.2824 |
|  | Arousal | -1.466 | 127 | .145 | -0.2957 |
| Safety reversal | SCR | 1.373 | 127 | .172 | 0.2770 |
|  | Valence | -0.216 | 127 | .829 | -0.0436 |
|  | Arousal | 0.497 | 127 | .620 | 0.1003 |

**Supplementary Table 13.** ANOVAs results for the effects of group and CS type on SCR, arousal and valence during trace conditioning

| ANOVA | Effect | *F* | *df* | *p* | η2 |
| --- | --- | --- | --- | --- | --- |
|  | G | <.01 | 1, 122 | .963 | <.001 |
| SCR | CS^a^ | 45.13 | 1.46, 178.4 | <.001 | .270 |
|  | CS X G^a^ | <.001 | 1.46, 178.4 | .997 | <.001 |
|  | G | 3.47 | 1, 122 | .065 | .028 |
| Arousal ratings | CS^b^ | 127.83 | 1.88, 229.47 | <.001 | .512 |
|  | CS X G^b^ | 2.26 | 1.88, 229.47 | .110 | .018 |
|  | G | 0.15 | 1, 122 | .699 | .001 |
| Valence ratings | CS^c^ | 111.68 | 1.69, 205.62 | <.001 | .478 |
|  | CS X G^c^ | 1.84 | 1.69, 205.62 | .167 | .015 |

*Note*: G = Group; CS = CS type. Greenhouse-Geisser corrected values identified with lowercase letters.

^a^: Mauchly’s W = .632, p < .001, Greenhouse-Geisser ε = .731

^b^: Mauchly’s W = .937, p = .019, Greenhouse-Geisser ε = .940

^c^: Mauchly’s W = .813, p < .001, Greenhouse-Geisser ε = .843

**Supplementary Table 14.** ANOVA results for the effects of group, CS type, and phase on SCR during trace fear conditioning.

| Effect | *F* | *df* | *p* | η2 |
| --- | --- | --- | --- | --- |
| G | 0.01 | 1, 122 | .963 | .000 |
| P | 76.83 | 1, 122 | <.001 | .386 |
| P x G | 0.39 | 1, 122 | .533 | .003 |
| CS^a^ | 45.13 | 1.46, 178.40 | <.001 | .270 |
| CS x G^a^ | 0.00 | 1.46, 178.40 | .997 | .000 |
| P x CS^b^ | 6.87 | 1.83, 222.98 | .002 | .053 |
| P x CS x G^b^ | 0.66 | 1.83, 222.98 | .506 | .005 |

*Note*: G = Group, P = Phase, CS = CS type. Greenhouse-Geisser corrected values identified with lowercase letters.

^a^: Mauchly’s W = .632, p < .001, Greenhouse-Geisser ε = .731

^b^: Mauchly’s W = .906, p = .003, Greenhouse-Geisser ε = .914

**Supplementary Table 15.** Brain activations during trace fear conditioning (CS50+ vs. CS-) for healthy controls.

| AAL Region | Cluster *p* value FWER | N voxels | *t* | MNI (x,y,z) | Peak *p* value uncorrected |
| --- | --- | --- | --- | --- | --- |
| Thalamus | <0.001 | 191 | 9.32 | 6, -21, -3 | <0.001 |
|  |  |  | 7.09 | -6, -21, -3 | <0.001 |
|  |  |  | 6.34 | 12, -18, 6 | <0.001 |
| Supp_Motor_Area | <0.001 | 609 | 7.86 | -9, -9, 69 | <0.001 |
|  |  |  | 7.72 | 9, -6, 69 | <0.001 |
|  |  |  | 7.58 | 6, 3, 51 | <0.001 |
| Precentral_L | <0.001 | 188 | 7.67 | -42, -6, 51 | <0.001 |
|  |  |  | 6.3 | -36, -18, 42 | <0.001 |
|  |  |  | 4.8 | -51, 0, 42 | <0.001 |
| SupraMarginal_L | <0.001 | 193 | 7.2 | -51, -33, 27 | <0.001 |
|  |  |  | 6.11 | -57, -39, 33 | <0.001 |
|  |  |  | 5.96 | -60, -21, 24 | <0.001 |
| Postcentral_R | <0.001 | 49 | 6.07 | 30, -27, 66 | <0.001 |
|  |  |  | 6.02 | 27, -27, 57 | <0.001 |
| Insula_L | <0.001 | 50 | 5.71 | -33, 27, 3 | <0.001 |
|  |  |  | 5.63 | -33, 18, 9 | <0.001 |
| Frontal_Mid_R | <0.001 | 23 | 5.65 | 45, -3, 54 | <0.001 |

*Note:* AAL = Automated Anatomical Labeling, FWER = Family Wise Error Rate, N = Number, MNI = Montreal Neurological Institute

**Supplementary Table 16.** Brain activations during trace fear conditioning (CS50+ vs. CS-) for patients with anxiety disorders.

| AAL Region | Cluster *p* value FWER | N voxels | *t* | MNI (x,y,z) | Peak *p* value uncorrected |
| --- | --- | --- | --- | --- | --- |
| Cingulum_Mid_R | <0.001 | 285 | 7.71 | 9, -27, 51 | <0.001 |
| Postcentral_R |  |  | 5.56 | 30, -30, 60 | <0.001 |
|  |  |  | 4.94 | 24, -36, 60 | <0.001 |
| Supp_Motor_Area_R | <0.001 | 583 | 6.94 | 3, -6, 60 | <0.001 |
|  |  |  | 6.06 | 3, 0, 72 | <0.001 |
|  |  |  | 5.86 | 9, 6, 57 | <0.001 |
| Precentral_L | <0.001 | 153 | 6.52 | -36, -6, 48 | <0.001 |
|  |  |  | 5.68 | -45, -6, 51 | <0.001 |
|  |  |  | 5.2 | -51, 3, 42 | <0.001 |
| SupraMarginal_L | <0.001 | 141 | 6.25 | -63, -27, 21 | <0.001 |
|  |  |  | 4.86 | -60, -18, 21 | <0.001 |
|  |  |  | 4.5 | -54, -42, 39 | <0.001 |
| Fusiform_R | <0.001 | 292 | 5.75 | 36, -75, -15 | <0.001 |
|  |  |  | 5.66 | 36, -84, -12 | <0.001 |
|  |  |  | 5.23 | -6, -87, 3 | <0.001 |
| SupraMarginal_R | <0.001 | 109 | 5.67 | 54, -27, 24 | <0.001 |
|  |  |  | 4.58 | 48, -15, 18 | <0.001 |
|  |  |  | 3.67 | 60, -39, 27 | <0.001 |
| Frontal_Inf_Oper_L | <0.001 | 247 | 5.48 | -57, 9, 15 | <0.001 |
|  |  |  | 5.14 | -51, 0, 6 | <0.001 |
| Insula_L |  |  | 5.01 | -42, 18, 0 | <0.001 |
| Insula_R | <0.05 | 59 | 5.18 | 36, 24, 0 | <0.001 |
| Frontal_Inf_Oper_R | <0.05 | 46 | 4.73 | 54, 15, 0 | <0.001 |
|  |  |  | 4.42 | 51, 6, 0 | <0.001 |
|  |  |  | 3.99 | 48, 0, 6 | <0.001 |

*Note:* AAL = Automated Anatomical Labeling, FWER = Family Wise Error Rate, N = Number, MNI = Montreal Neurological Institute

**Supplementary Table 17**. Brain activations during trace fear conditioning (CS81+ vs. CS-) for healthy controls.

| AAL Region | Cluster *p* value FWER | N voxels | *t* | MNI (x,y,z) | Peak *p* value uncorrected |
| --- | --- | --- | --- | --- | --- |
| Thalamus | <0.001 | 439 | 10.08 | 6, -21, 0 | <0.001 |
|  |  |  | 9.35 | -15, -18, 6 | <0.001 |
|  |  |  | 8.64 | -6, -21, -3 | <0.001 |
| Precentral_L | <0.001 | 197 | 8.79 | -39, -9, 48 | <0.001 |
|  |  |  | 5.49 | -48, 3, 45 | <0.001 |
| SupraMarginal_L | <0.001 | 276 | 8.72 | -51, -33, 27 | <0.001 |
|  |  |  | 6.58 | -45, -27, 9 | <0.001 |
|  |  |  | 6.34 | -54, -18, 24 | <0.001 |
| Insula_L | <0.001 | 147 | 8.04 | -30, 27, 3 | <0.001 |
|  |  |  | 7.82 | -33, 18, 9 | <0.001 |
|  |  |  | 5.45 | -51, 6, 3 | <0.001 |
| Supp_Motor_Area | <0.001 | 403 | 7.99 | 6, -18, 54 | <0.001 |
|  |  |  | 7.57 | 6, -3, 57 | <0.001 |
|  |  |  | 7.06 | -3, 3, 54 | <0.001 |
| Postcentral_R | <0.001 | 104 | 7.75 | 33, -30, 63 | <0.001 |
|  |  |  | 7.56 | 27, -27, 54 | <0.001 |
| Caudate_L | <0.001 | 28 | 7.04 | -9, 6, 6 | <0.001 |
| Insula_R | <0.001 | 52 | 6.86 | 33, 27, 0 | <0.001 |
|  |  |  | 6.31 | 33, 24, 9 | <0.001 |
| SupraMarginal_R | <0.001 | 20 | 5.83 | 54, -33, 30 | <0.001 |
| Putamen_R | <0.001 | 20 | 5.65 | 30, 0, 0 | <0.001 |

*Note:* AAL = Automated Anatomical Labeling, FWER = Family Wise Error Rate, N = Number, MNI = Montreal Neurological Institute

**Supplementary Table 18**. Brain activations during trace fear conditioning (CS81+ vs. CS-) for patients with anxiety disorders.

| AAL Region | Cluster *p* value FWER | N voxels | *t* | MNI (x,y,z) | Peak *p* value uncorrected |
| --- | --- | --- | --- | --- | --- |
| Supp_Motor_Area | <0.001 | 460 | 8.94 | 0, -6, 60 | <0.001 |
|  |  |  | 7.49 | 6, 6, 54 | <0.001 |
|  |  |  | 6.67 | 6, -27, 54 | <0.001 |
| Insula_L | <0.001 | 253 | 7.61 | -36, 24, 3 | <0.001 |
|  |  |  | 5.55 | -57, 6, 12 | <0.001 |
|  |  |  | 4.67 | -51, 3, 3 | <0.001 |
| Thalamus | <0.001 | 99 | 6.76 | 3, -21, -6 | <0.001 |
|  |  |  | 5.38 | 9, -15, -6 | <0.001 |
|  |  |  | 5 | -9, -18, 9 | <0.001 |
| Precentral_L | <0.001 | 442 | 6.71 | -36, -6, 51 | <0.001 |
|  |  |  | 6.68 | -42, -12, 54 | <0.001 |
|  |  |  | 5.63 | -51, -3, 42 | <0.001 |
| Parietal_Inf_L | <0.001 | 303 | 6.51 | -54, -39, 39 | <0.001 |
| SupraMarginal_L |  |  | 5.39 | -60, -33, 27 | <0.001 |
|  |  |  | 5.32 | -51, -27, 15 | <0.001 |
| Cingulum_Mid | <0.01 | 79 | 5.88 | -12, -24, 45 | <0.001 |
|  |  |  | 4 | -15, -39, 51 | <0.001 |
| Precentral_R | <0.001 | 205 | 5.62 | 33, -27, 63 | <0.001 |
|  |  |  | 5.25 | 42, -12, 57 | <0.001 |
|  |  |  | 4.92 | 33, -36, 63 | <0.001 |
| Insula_R | <0.001 | 183 | 5.58 | 39, 21, 6 | <0.001 |
|  |  |  | 5.21 | 48, 6, 3 | <0.001 |
|  |  |  | 4.93 | 54, 12, 9 | <0.001 |
| SupraMarginal_R | <0.01 | 95 | 5.55 | 51, -30, 27 | <0.001 |
|  |  |  | 5.03 | 45, -24, 21 | <0.001 |
| Calcarine | <0.001 | 193 | 5.09 | -9, -90, 3 | <0.001 |
|  |  |  | 5.04 | 3, -81, 3 | <0.001 |
|  |  |  | 4.82 | 9, -87, 6 | <0.001 |

*Note:* AAL = Automated Anatomical Labeling, FWER = Family Wise Error Rate, N = Number, MNI = Montreal Neurological Institute

**Supplementary Table 19**. Brain activations during trace fear conditioning (CS81+ vs. CS50+) for healthy controls.

| AAL Region | Cluster *p* value FWER | N voxels | *t* | MNI (x,y,z) | Peak *p* value uncorrected |
| --- | --- | --- | --- | --- | --- |
| Temporal_Mid_R | <0.001 | 371 | 4.9 | 39, -51, 15 | <0.001 |
|  |  |  | 4.63 | 33, -60, 15 | <0.001 |
|  |  |  | 4.41 | 51, -57, 15 | <0.001 |
| Putamen_R | <0.01 | 142 | 4.89 | 30, -6, 0 | <0.001 |
|  |  |  | 4.74 | 24, -6, 9 | <0.001 |
| Hippocampus_R |  |  | 4.34 | 30, -24, -3 | <0.001 |
| Occipital_Mid_L | <0.001 | 716 | 4.46 | -39, -63, 18 | <0.001 |
|  |  |  | 4.37 | -36, -36, 15 | <0.001 |
| Putamen_L |  |  | 4.36 | -21, -9, 15 | <0.001 |
| Thalamus_L |  |  | 4.16 | -11, -22, 6 | <0.001 |
| Precentral_R | <0.05 | 79 | 4.43 | 36, -18, 48 | <0.001 |
| Postcentral_R |  |  | 4.3 | 30, -27, 54 | <0.001 |

*Note:* AAL = Automated Anatomical Labeling, FWER = Family Wise Error Rate, N = Number, MNI = Montreal Neurological Institute

**Additional analysis - Covariating for biological sex and age**

**Supplementary Table 20.** ANCOVA results for the effects group and CS type on SCR, arousal, and valence, covariating for biological sex and age during delay conditioning

| ANCOVA | Effect | *F* | *df* | *p* | η2 |
| --- | --- | --- | --- | --- | --- |
| SCR | G | 5.86 | 1, 124 | .017 | .045 |
|  | S | 4.00 | 1, 124 | .048 | .031 |
|  | A | 0.29 | 1, 124 | .594 | .002 |
|  | G x S | 0.45 | 1, 124 | .505 | .004 |
|  | CS | 6.62 | 1, 124 | .011 | .051 |
|  | CS x G | 0.60 | 1, 124 | .439 | .005 |
|  | CS x S | 0.03 | 1, 124 | .866 | <.001 |
|  | CS x A | 0.82 | 1, 124 | .368 | .007 |
|  | CS x G x S | 3.88 | 1, 124 | .051 | .030 |
| Arousal ratings | G | 1.46 | 1, 124 | .229 | .012 |
|  | S | 1.51 | 1, 124 | .221 | .012 |
|  | A | 0.08 | 1, 124 | .778 | <.001 |
|  | G x S | 2.92 | 1, 124 | .090 | .023 |
|  | CS | 20.53 | 1, 124 | <.001 | .142 |
|  | CS x G | 0.20 | 1, 124 | .659 | .002 |
|  | CS x S | 3.55 | 1, 124 | .062 | .028 |
|  | CS x A | 1.63 | 1, 124 | .205 | .013 |
|  | CS x G x S | 3.30 | 1, 124 | .071 | .026 |
| Valence ratings | G | 0.05 | 1, 124 | .822 | <.001 |
|  | S | 0.04 | 1, 124 | .851 | <.001 |
|  | A | 1.11 | 1, 124 | .293 | .009 |
|  | G x S | 0.09 | 1, 124 | .767 | <.001 |
|  | CS | 13.68 | 1, 124 | <.001 | .099 |
|  | CS x G | 0.16 | 1, 124 | .693 | <.001 |
|  | CS x S | 7.39 | 1, 124 | .007 | .056 |
|  | CS x A | 0.21 | 1, 124 | .651 | <.001 |
|  | CS x G x S | 2.69 | 1, 124 | .104 | .021 |

*Note*: G=Group, S=Sex, A=Age, CS = CS type

**Supplementary Table 21.** ANCOVA results for the effects of group and CS type on SCR, arousal, and valence, covariating for biological sex and age during fear reversal.

| ANCOVA | Effect | *F* | *df* | *p* | η2 |
| --- | --- | --- | --- | --- | --- |
| SCR | G | 1.46 | 1, 124 | .228 | .012 |
|  | S | 0.03 | 1, 124 | .867 | <.001 |
|  | A | 0.02 | 1, 124 | .877 | <.001 |
|  | G x S | 1.06 | 1, 124 | .305 | .008 |
|  | CS | 6.41 | 1, 124 | .013 | .049 |
|  | CS x G | 0.64 | 1, 124 | .425 | .005 |
|  | CS x S | 0.31 | 1, 124 | .579 | .002 |
|  | CS x A | 0.54 | 1, 124 | .463 | .004 |
|  | CS x G x S | 0.95 | 1, 124 | .331 | .008 |
| Arousal ratings | G | 2.65 | 1, 124 | .106 | .021 |
|  | S | 0.00 | 1, 124 | .946 | <.001 |
|  | A | 2.64 | 1, 124 | .107 | .021 |
|  | G x S | 1.12 | 1, 124 | .293 | .009 |
|  | CS | 17.62 | 1, 124 | <.001 | .124 |
|  | CS x G | 1.83 | 1, 124 | .179 | .015 |
|  | CS x S | 0.16 | 1, 124 | .687 | .001 |
|  | CS x A | 1.69 | 1, 124 | .196 | .013 |
|  | CS x G x S | 2.22 | 1, 124 | .139 | .018 |
| Valence ratings | G | 0.47 | 1, 124 | .492 | .004 |
|  | S | 1.06 | 1, 124 | .306 | .008 |
|  | A | 0.71 | 1, 124 | .401 | .006 |
|  | G x S | 3.13 | 1, 124 | .079 | .025 |
|  | CS | 12.88 | 1, 124 | <.001 | .094 |
|  | CS x G | 1.33 | 1, 124 | .252 | .011 |
|  | CS x S | 0.10 | 1, 124 | .758 | <.001 |
|  | CS x A | 1.19 | 1, 124 | .277 | .010 |
|  | CS x G x S | 0.89 | 1, 124 | .347 | .007 |

*Note*: G=Group, S=Sex, A=Age, CS = CS type

**Supplementary Table 22**. ANCOVA results for the effects of group and CS type on SCR, arousal, and valence, covariating for biological sex and age during trace conditioning.

| ANCOVA | Effect | *F* | *df* | *p* | η2 |
| --- | --- | --- | --- | --- | --- |
| SCR | G | 0.06 | 1, 119 | .805 | .001 |
|  | S | 0.00 | 1, 119 | .979 | .000 |
|  | A | 2.83 | 1, 119 | .095 | .023 |
|  | G x S | 3.65 | 1, 119 | .058 | .030 |
|  | CS^a^ | 6.75 | 1.47, 175.26 | .004 | .054 |
|  | CS x G^a^ | 0.01 | 1.47, 175.26 | .975 | .000 |
|  | CS x S^a^ | 1.54 | 1.47, 175.26 | .220 | .013 |
|  | CS x A^a^ | 1.59 | 1.47, 175.26 | .210 | .013 |
|  | CS x G x S^a^ | 2.40 | 1.47, 175.26 | .109 | .020 |
| Arousal Ratings | G | 3.79 | 1, 119 | .054 | .031 |
|  | S | 5.12 | 1, 119 | .026 | .041 |
|  | A | 0.23 | 1, 119 | .636 | .002 |
|  | G x S | 1.20 | 1, 119 | .275 | .010 |
|  | CS^b^ | 12.04 | 1.90, 225.95 | <.001 | .092 |
|  | CS x G^b^ | 2.19 | 1.90, 225.95 | .117 | .018 |
|  | CS x S^b^ | 4.72 | 1.90, 225.95 | .011 | .038 |
|  | CS x A^b^ | 1.55 | 1.90, 225.95 | .215 | .013 |
|  | CS x G x S^b^ | 2.43 | 1.90, 225.95 | .093 | .020 |
| Valence Ratings | G | 0.18 | 1, 119 | .671 | .002 |
|  | S | 1.29 | 1, 119 | .258 | .011 |
|  | A | 0.42 | 1, 119 | .519 | .004 |
|  | G x S | 0.08 | 1, 119 | .775 | .001 |
|  | CS^c^ | 18.36 | 1.72, 204.34 | <.001 | .134 |
|  | CS x G^c^ | 1.92 | 1.72, 204.34 | .156 | .016 |
|  | CS x S^c^ | 1.13 | 1.72, 204.34 | .320 | .009 |
|  | CS x A^c^ | 4.81 | 1.72, 204.34 | .013 | .039 |
|  | CS x G x S^c^ | 0.12 | 1.72, 204.34 | .857 | .001 |

*Note*: G=Group, S=Sex, A=Age, CS = CS type. Greenhouse-Geisser corrected values identified with lowercase letters.

^a^: Mauchly’s W = .642, p < .001, Greenhouse-Geisser ε = .736

^b^: Mauchly’s W = .947, p = .039, Greenhouse-Geisser ε = .949

^c^: Mauchly’s W = .835, p < .001, Greenhouse-Geisser ε = .859

**Additional analyses. Adding task order as factor**

**Supplementary Table 23.** Significant differences in brain activation during early delay fear conditioning (CS+>CS-) between patients with anxiety disorders and healthy controls when task order was included as a factor.

| AAL Region | Cluster *p* value FWER | N voxels | *t* | MNI (x,y,z) | Peak *p* value uncorrected |
| --- | --- | --- | --- | --- | --- |
| Frontal_Mid_L | <0.05 | 70 | 3.99 | -27, 33, 21 | <0.001 |
|  |  |  | 3.69 | -30, 45, 12 | <0.001 |

*Note:* AAL = Automated Anatomical Labeling, FWER = Family Wise Error Rate, N = Number, MNI = Montreal Neurological Institute

**Supplementary Table 24**: ANOVA results for the effects of group, CS type, and task order (delayed-reversal first versus trace first) on SCR during delay fear conditioning.

| ANOVA | Effect | F | DF | p | Eta2p |
| --- | --- | --- | --- | --- | --- |
|  | G | 6.07 | 1, 125 | .015 | .046 |
|  | TO | .52 | 1, 125 | .472 | .004 |
|  | G x TO | .09 | 1, 125 | .771 | .001 |
| SCR | CS | 71.78 | 1, 125 | <.001 | .365 |
|  | CS x G | .33 | 1, 125 | .568 | .003 |
|  | CS x TO | .17 | 1, 125 | .685 | .001 |
|  | CS x G x TO | .01 | 1, 125 | .928 | <.001 |
|  | G | 2.16 | 1, 125 | .145 | .017 |
|  | TO | 3.12 | 1, 125 | .080 | .024 |
|  | G x TO | 2.79 | 1, 125 | .100 | .022 |
| Arousal ratings | CS | 276.83 | 1, 125 | <.001 | .689 |
|  | CS x G | .34 | 1, 125 | .563 | .003 |
|  | CS x TO | 1.25 | 1, 125 | .265 | .010 |
|  | CS x G x TO | .01 | 1, 125 | .942 | <.001 |
|  | G | .04 | 1, 125 | .849 | <.001 |
|  | TO | 3.17 | 1, 125 | .077 | .025 |
|  | G x TO | 1.29 | 1, 125 | .258 | .010 |
| Valence ratings | CS | 274.85 | 1, 125 | <.001 | .687 |
|  | CS x G | .24 | 1, 125 | .622 | .002 |
|  | CS x TO | 3.26 | 1, 125 | .074 | .025 |
|  | CS x G x TO | .25 | 1, 125 | .619 | .002 |

Note: G = Group; TO = Task Order; CS = CS type.

**Supplementary Table 25**: ANOVA results for the effects of group, CS type, and task order (delayed-reversal first versus trace first) on SCR during reversal conditioning.

| ANOVA | Effect | F | DF | p | Eta2p |
| --- | --- | --- | --- | --- | --- |
|  | G | 1.71 | 1, 125 | .193 | .014 |
|  | TO | 1.44 | 1, 125 | .233 | .011 |
|  | G x TO | .56 | 1, 125 | .455 | .004 |
| SCR | CS | 96.20 | 1, 125 | <.001 | .408 |
|  | CS x G | .776 | 1, 125 | .380 | .006 |
|  | CS x TO | 1.41 | 1, 125 | .237 | .011 |
|  | CS x G x TO | .09 | 1, 125 | .763 | .001 |
|  | G | 3.20 | 1, 125 | .076 | .025 |
|  | TO | 4.92 | 1, 125 | .028 | .038 |
|  | G x TO | .03 | 1, 125 | .860 | <.001 |
| Arousal ratings | CS | 218.95 | 1, 125 | <.001 | .637 |
|  | CS x G | 2.23 | 1, 125 | .138 | .017 |
|  | CS x TO | .23 | 1, 125 | .636 | .002 |
|  | CS x G x TO | .17 | 1, 125 | .685 | .001 |
|  | G | .69 | 1, 125 | .408 | .005 |
|  | TO | 2.98 | 1, 125 | .087 | .023 |
|  | G x TO | .44 | 1, 125 | .509 | .003 |
| Valence ratings | CS | 164.53 | 1, 125 | <.001 | .568 |
|  | CS x G | 1.02 | 1, 125 | .314 | .008 |
|  | CS x TO | 1.74 | 1, 125 | .189 | .014 |
|  | CS x G x TO | .374 | 1, 125 | .542 | .003 |

Note: G = Group; TO = Task Order; CS = CS type.

**Supplementary Table 26.** ANOVA results for the effects of group, CS type, and task order (delayed-reversal first versus trace first) on SCR during trace fear conditioning.

| ANOVA | Effect | F | DF | p | Eta2p |
| --- | --- | --- | --- | --- | --- |
|  | G | <.001 | 1, 120 | .980 | <.001 |
|  | TO | .14 | 1, 120 | .710 | .001 |
|  | G x TO | .004 | 1, 120 | .953 | <.001 |
| SCR | CS^a^ | 43.65 | 1.46, 175.15 | <.001 | .266 |
|  | CS x G^a^ | .002 | 1.46, 175.15 | .990 | <.001 |
|  | CS x TO^a^ | .03 | 1.46, 175.15 | .933 | <.001 |
|  | CS x G x TO^a^ | .15 | 1.46, 175.15 | .795 | .001 |
|  | G | 3.57 | 1, 120 | .061 | .029 |
|  | TO | .11 | 1, 120 | .740 | <.001 |
|  | G x TO | .89 | 1, 120 | .346 | .007 |
| Arousal ratings | CS^b^ | 127.85 | 1.86, 223.08 | <.001 | .516 |
|  | CS x G^b^ | 2.56 | 1.86, 223.08 | .084 | .021 |
|  | CS x TO^b^ | 2.49 | 1.86, 223.08 | .090 | .020 |
|  | CS x G x TO^b^ | 2.08 | 1.86, 223.08 | .131 | .017 |
|  | G | .06 | 1, 120 | .801 | <.001 |
|  | TO | 4.92 | 1, 120 | .028 | .039 |
|  | G x TO | .01 | 1, 120 | .920 | <.001 |
| Valence ratings | CS^c^ | 108.14 | 1.70, 203.96 | <.001 | .474 |
|  | CS x G^c^ | 2.44 | 1.70, 203.96 | .098 | .020 |
|  | CS x TO^c^ | 4.71 | 1.70, 203.96 | .014 | .038 |
|  | CS x G x TO^c^ | 1.66 | 1.70, 203.96 | .196 | .014 |

Note: G = Group; TO = Task Order; CS = CS type. Greenhouse-Geisser corrected values identified with lowercase letters.

a: Mauchly’s W = .630, p < .001, Greenhouse-Geisser ε = .730

b: Mauchly’s W = .924, p = .009, Greenhouse-Geisser ε = .930

c: Mauchly’s W = .823, p < .001, Greenhouse-Geisser ε = .850

**SUPPLEMENTARY FIGURES**

**Delay Fear Conditioning**

Preconditioning


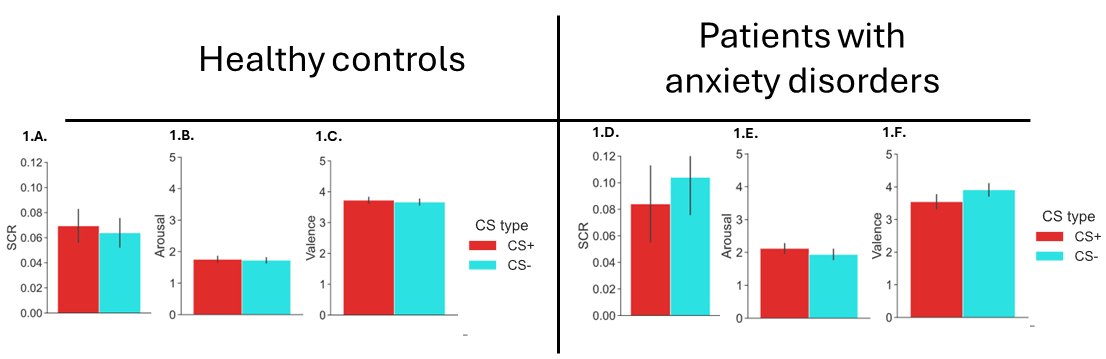


**Supplementary Figure 1**: Responses during preconditioning in the delay conditioning/reversal task in healthy controls (n=98) and patients with anxiety disorders (n=33). LEFT: SCR (last trial) (A), subjective ratings of arousal (B) and valence (C) RIGHT: SCR (last trial) (D) subjective ratings of arousal (E) and valence (F). SCR = skin conductance response. CS = conditioned stimuli. Error bars indicate standard error of the mean (SEM).

**Trace Fear Conditioning**


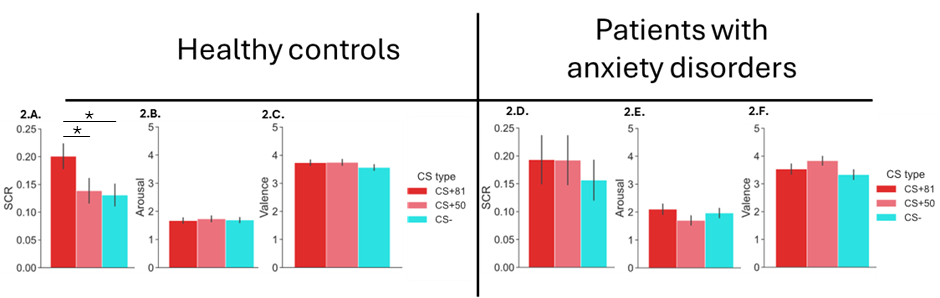
Preconditioning

**Supplementary Figure 2**: Responses during preconditioning in the trace conditioning task in healthy controls (n=93) and patients with anxiety disorders (n=31). LEFT: SCR (last trial) (A), subjective ratings of arousal (B) and valence (C) RIGHT: SCR (last trial) (D) subjective ratings of arousal (E) and valence (F). SCR = skin conductance response. CS = conditioned stimuli. Error bars indicate standard error of the mean (SEM). **p<0.01.*

Conditioning – contrast CS81+ > CS50+

**
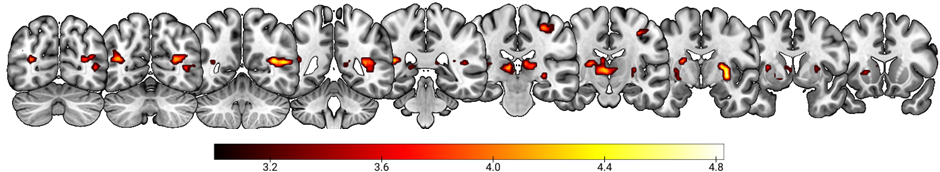
Supplementary Figure 3.** Clusters of fMRI activations (CS81+ > CS50+) for trace conditioning in healthy controls

**SUPPLEMENTARY REFERENCES**

1 Buela-Casal G, Guillén-Riquelme A, Seisdedos Cubero N. *Cuestionario de Ansiedad Estado-Rasgo*. 9th ed. TEA Ediciones: Madrid, 2016.

2 Spielberg CD, Gorsuch RL,, Lushene RE. *Manual for the State-Trait Inventory*. Consulting Psychological Press: Palo Alto: CA, 1970.

3 Ferrando L, Franco A, Soto M, Bobes J, Soto O, Franco L *et al.* *MINI Entrevista Neuropsiquiátrica Internacional*. Instituto IAP: Madrid, 1998.

4 Sheehan D V, Lecrubier Y, Sheehan KH, Amorim P, Janavs J, Weiller E *et al.* The Mini-International Neuropsychiatric Interview (M.I.N.I.): the development and validation of a structured diagnostic psychiatric interview for DSM-IV and ICD-10. *J Clin Psychiatry* 1998; 59 Suppl 20: 22–33.

5 Freeston MH, Rheaume J, Letarte H, Dugas MJ, Ladouceur R. Intolerance of Uncertainty Scale. *PsycTESTS Dataset* 2012. doi:10.1037/T01110-000.

6 González Rodríguez M, Cubas León R, Rovella AT, Darias Herrera M. Adaptación española de la escala de intolerancia hacia la incertidumbre: procesos cognitivos, ansiedad y depresión. *Psicología y Salud* 2006; 16: 219–233.

7 Bobes J, Badía X, Luque A, García M, González MP,, Dal-Ré R,. Validation of the Spanish version of the Liebowitz social anxiety scale, social anxiety and distress scale and Sheehan disability inventory for the evaluation of social phobia. *Medicina Clínica (Barcelona)* 1999; 112: 130–138.

8 Liebowitz MR. Social Phobia. *Mod Probl Pharmacopsychiatry* 1987; : 141–173.

9 Bobes J, García-Calvo C, Prieto R, García-García M, Rico-Villademoros F,, Grupo Español de Trabajo para la validación de la versión española de la Escala de Detección del Trastorno de Ansiedad Generalizada según DSM-IV (Escala de TAG de Carrol y Davidson)). Psychometric properties of the Spanish version of the screening scale for DSM-IV Generalized Anxiety Disorder of Carroll and Davidson. *Actas Esp Psiquiatr* 2006; 34: 83–93.

10 Carroll BJ, Davidson JRT. Screening scale for DSM-IV GAD. *Copyright* 2000.

11 Meyer TJ, Miller ML, Metzger RL, Borkovec T. Development and validation of the Penn State Worry Questionnaire. *Behaviour research and therapy* 1990; 28: 487–495.

12 Sandín B, Chorot P, Valiente RM, Lostao L. Validación española del cuestionario de preocupación PSWQ : estructura factorial y propiedades psicométricas. *Revista de Psicopatología y Psicología Clínica* 2009; 14: 107–122.

13 Bados A, Solanas A, Andrés R. Psychometric properties of the Spanish version of Depression, Anxiety and Stress Scales (DASS). *Psicothema* 2005; 17: 679–683.

14 Lovibond SH, Lovibond PF. Depression Anxiety Stress Scales. *PsycTESTS Dataset* 1995. doi:10.1037/T01004-000.

15 Tustison NJ, Avants BB, Cook PA, Yuanjie Zheng, Egan A, Yushkevich PA *et al.* N4ITK: Improved N3 Bias Correction. *IEEE Trans Med Imaging* 2010; 29: 1310–1320.

16 Jenkinson M, Beckmann CF, Behrens TEJ, Woolrich MW, Smith SM. FSL. *Neuroimage* 2012; 62: 782–790.

17 Dale AM, Fischl B, Sereno MI. Cortical Surface-Based Analysis: I. Segmentation and Surface Reconstruction. *NeuroImage (Orlando, Fla)* 1999; 9: 179–194.

18 Klein A, Ghosh SS, Bao FS, Giard J, Häme Y, Stavsky E *et al.* Mindboggling morphometry of human brains. *PLoS Comput Biol* 2017; 13: e1005350–e1005350.

19 Fonov V, Evans A, McKinstry R, Almli C, Collins D. Unbiased nonlinear average age-appropriate brain templates from birth to adulthood. *NeuroImage (Orlando, Fla)* 2009; 47: S102–S102.

20 Esteban O, Ciric R, Finc K, Blair RW, Markiewicz CJ, Moodie CA *et al.* Analysis of task-based functional MRI data preprocessed with fMRIPrep. *Nat Protoc* 2020; 15: 2186–2202.

21 Gorgolewski K, Burns CD, Madison C, Clark D, Halchenko YO, Waskom ML *et al.* Nipype: a flexible, lightweight and extensible neuroimaging data processing framework in python. *Front Neuroinform* 2011; 5: 13–13.

22 Greve DN, Fischl B. Accurate and robust brain image alignment using boundary-based registration. *NeuroImage (Orlando, Fla)* 2009; 48: 63–72.

23 Cox RW, Hyde JS. Software tools for analysis and visualization of fMRI data. *NMR Biomed* 1997; 10: 171–178.

24 Jenkinson M, Bannister P, Brady M, Smith S. Improved Optimization for the Robust and Accurate Linear Registration and Motion Correction of Brain Images. *NeuroImage (Orlando, Fla)* 2002; 17: 825–841.

25 Lanczos C. Evaluation of Noisy Data. *Journal of the Society for Industrial and Applied Mathematics Series B, Numerical analysis* 1964; 1: 76–85.

26 Pruim RHR, Mennes MJJ, Rooij D van, Llera A, Buitelaar JK, Beckmann CF. ICA-AROMA: A robust ICA-based strategy for removing motion artifacts from fMRI data. *NeuroImage (Orlando, Fla)* 2015; 112: 267–277.

27 Behzadi Y, Restom K, Liau J, Liu TT. A component based noise correction method (CompCor) for BOLD and perfusion based fMRI. *NeuroImage (Orlando, Fla)* 2007; 37: 90–101.

28 Power JD, Mitra A, Laumann TO, Snyder AZ, Schlaggar BL, Petersen SE. Methods to detect, characterize, and remove motion artifact in resting state fMRI. *NeuroImage (Orlando, Fla)* 2014; 84: 320–341.
